# SAFit2 reduces neuroinflammation and ameliorates nerve injury-induced neuropathic pain

**DOI:** 10.1186/s12974-022-02615-7

**Published:** 2022-10-10

**Authors:** Saskia Wedel, Praveen Mathoor, Oliver Rauh, Tim Heymann, Cosmin I. Ciotu, Dominik C. Fuhrmann, Michael J. M. Fischer, Andreas Weigert, Natasja de Bruin, Felix Hausch, Gerd Geisslinger, Marco Sisignano

**Affiliations:** 1 Institute of Clinical Pharmacology, Pharmazentrum Frankfurt/ZAFES, University Hospital, Goethe-University, 60590 Frankfurt am Main, Germany; 2 Institute of Biochemistry I, Faculty of Medicine, Goethe-University Frankfurt, 60590 Frankfurt am Main, Germany; 3 Membrane Biophysics, Department of Biology, Technical University of Darmstadt, 64287 Darmstadt, Germany; 4 Department of Chemistry, Technical University of Darmstadt, 64287 Darmstadt, Germany; 5 Center of Physiology and Pharmacology, Medical University of Vienna, 1090 Vienna, Austria; 6 Fraunhofer Institute for Translational Medicine and Pharmacology ITMP and Fraunhofer Cluster of Excellence for Immune Mediated Diseases CIMD, 60596 Frankfurt am Main, Germany

**Keywords:** SAFit2, FKBP51, Neuropathic pain, Neuroinflammation, Sensory neurons

## Abstract

**Background:**

Neuropathic pain is experienced worldwide by patients suffering from nerve injuries, infectious or metabolic diseases or chemotherapy. However, the treatment options are still limited because of low efficacy and sometimes severe side effects. Recently, the deficiency of FKBP51 was shown to relieve chronic pain, revealing FKBP51 as a potential therapeutic target. However, a specific and potent FKBP51 inhibitor was not available until recently which hampered targeting of FKBP51.

**Methods:**

In this study, we used the well-established and robust spared nerve injury model to analyze the effect of SAFit2 on nerve injury-induced neuropathic pain and to elucidate its pharmacodynamics profile. Therefore, the mice were treated with 10 mg/kg SAFit2 after surgery, the mice behavior was assessed over 21 days and biochemical analysis were performed after 14 and 21 days. Furthermore, the impact of SAFit2 on sensory neurons and macrophages was investigated in vitro.

**Results:**

Here, we show that the FKBP51 inhibitor SAFit2 ameliorates nerve injury-induced neuropathic pain in vivo by reducing neuroinflammation. SAFit2 reduces the infiltration of immune cells into neuronal tissue and counteracts the increased NF-κB pathway activation which leads to reduced cytokine and chemokine levels in the DRGs and spinal cord. In addition, SAFit2 desensitizes the pain-relevant TRPV1 channel and subsequently reduces the release of pro-inflammatory neuropeptides from sensory neurons.

**Conclusions:**

SAFit2 ameliorates neuroinflammation and counteracts enhanced neuronal activity after nerve injury leading to an amelioration of nerve injury-induced neuropathic pain. Based on these findings, SAFit2 constitutes as a novel and promising drug candidate for the treatment of nerve injury-induced neuropathic pain.

**Supplementary Information:**

The online version contains supplementary material available at 10.1186/s12974-022-02615-7.

## Background

Neuropathic pain is defined as lesion or disease of the somatosensory system [8]. It is a pathological pain state experienced worldwide by patients suffering from nerve injuries, diseases such as diabetes or chemotherapy [50]. However, the therapeutic options for neuropathic pain are very limited due to low treatment responses and severe side effects of available drugs [12]. Nerve injury-induced neuropathic pain comprises several types of symptoms such as mechanical hypersensitivity which is defined as the reduction of the mechanical pain threshold, and painful sensations that can be mediated by innocuous stimuli [60].

The pain state itself arises by an increased activity of damaged nerve fibers, often in combination with an inflammatory response of the organism, which results in a pathological neuroinflammation [11]. Neuroinflammation is characterized as the infiltration of immune cells into neuronal tissue and the secretion of pro-inflammatory and proalgesic mediators such as cytokines, chemokines and neuropeptides that directly affect sensory neurons [22]. Moreover, the activation and sensitization of pain-mediating transient receptor potential (TRP) ion channels lead to the release of neuroinflammation-promoting neuropeptides [23].

During nerve injury-induced neuropathic pain, the release of pro-inflammatory and proalgesic mediators changes the microenvironment of sensory neurons which enhances neuronal activity. This can lead to peripheral and central sensitization and can subsequently to an increased pain perception [22]. The multitude of responsible mediators and the variety of different immune cell types, involved in their orchestrated synthesis and release, makes it difficult to target neuroinflammation at a large scale. Unfortunately, inhibiting individual cytokines, such as the tumor necrosis factor alpha, interleukin 6 or blocking their respective receptors does not significantly ameliorate neuropathic pain in patients [27, 58].

However, neuroinflammation can also be initiated by neurons themselves. More specifically, enhanced neuronal activity caused, for example, by increased activity of ion channels and depolarization can lead to an augmented downstream release of pro-inflammatory neuropeptides, such as calcitonin gene-related peptide (CGRP). These neuropeptides can cause vasodilation and recruitment of T cells and initiate neuroinflammation via their G protein-coupled receptors [3, 15, 56]. These studies indicate that a broader approach is required to target neuroinflammation-mediated persistent pain in patients.

Interestingly, previous studies revealed the FK506 binding protein 51 (FKBP51, encoded by the FKBP5 gene) as a novel potential therapeutic target for relieving neuropathic pain since its deficiency led to a significant amelioration of inflammatory and neuropathic pain [30, 31]. Initially, FKBP51 was discovered as a potential target for treating stress endocrinology [52] and glucocorticoid signaling related diseases [17]. Nevertheless, its strong upregulation, exclusively in sensory neurons of the dorsal horn after an ankle joint inflammation, indicated its involvement in the pathophysiological progress leading to chronic pain [31]. However, the pharmacological targeting of FKBP51 inhibitor was not possible for a long time due to low specificity of synthesized inhibitors and cross-interactions with other related proteins.

Nevertheless, these challenges were overcome, exploiting an FKBP51-specific conformation [20], to generate an highly potent FKBP51 inhibitor called SAFit2 (selective antagonist of FKBP51 by induced fit) [2, 13]. In addition, a good pharmacokinetic availability and an improved blood–brain barrier permeability of SAFit2 compared to SAFit1 was already confirmed in previous pharmacokinetic studies for the dose of 10 mg/kg SAFit2 injected intraperitoneally [13, 14]. However, the underlying analgesic and anti-hyperalgesic mechanisms have not been identified yet. Here, we used the well-established and robust spared nerve injury (SNI) mouse model to analyze the effect of SAFit2 on nerve injury-induced neuropathic pain. Further, we investigated the neuronal and neuroinflammatory effects of a SAFit2 treatment in vivo and in vitro to elucidate its pharmacodynamics profile.

## Materials and methods

### Animals, spared nerve injury surgery and SAFit2 treatment

In all experiments, wild-type mice (male C57Bl/6N mice, age from 8 to 12 weeks at the start of the study) were purchased from commercial breeding companies (Janvier and Charles River). For inducing neuropathic pain in mice, a spared nerve injury (SNI) surgery was performed under anesthesia to establish neuroinflammation. During the surgery, the sciatic nerve was exposed by a blunt dissection on the level of the knee joint. Then, two of the three sciatic nerve branches, common peroneal and tibial branch, were ligated with 6/0 non-sterile silk thread and cut distally from the ligature, leaving the sural nerve branch intact. Contact with the sural nerve branch was avoided to prevent stretching or harming. Afterwards, muscle and skin were closed in two layers [9].

For assessing the impact of SAFit2 on neuropathic pain behavior and neuroinflammation, animals were treated intraperitoneally with either 10 mg/kg SAFit2 or vehicle (PBS supplemented with 5% PEG400, 5% Tween and 0.7% ethanol) two times daily on six consecutive days starting on day five after surgery.

### Behavioral experiments

During all behavioral experiments, the experimenter was blinded. The motoric function of all animals was verified via rotarod before treatment. All animals were transferred into respective test cages for at least one hour before the measurement to allow habituation. For the determination of the mechanical withdrawal threshold, a dynamic plantar test was performed, using a Dynamic Plantar Aesthesiometer (Ugo Basile) as described previously. Briefly, a steel rod was pushed against the mid-plantar area of the hind paw with linear ascending force up to 5 g over 10 s and a holding force of 5 g until a withdrawal response occurred. For behavioral experiments a cut-off time of 20 s was set [49].

### Tissue isolation

For tissue isolation, mice were euthanized by isoflurane, cardio puncture and cranial dislocation either on day 14 or on day 21 after surgery covering different time points in neuroinflammation. The sciatic nerve, lumbar (L4–L6) dorsal root ganglia (DRGs) and the respective segments of the spinal cord were dissected from injured (ipsilateral) and unimpaired (contralateral) sites, followed by freezing tissue samples in liquid nitrogen for either RNA isolation, Western blot or multiplex assay. For flow cytometry analysis, the tissue was dissected and stored in 500 µl ice-cooled PBS at 4 °C until further processing.

### Quantitative real-time PCR

Total RNA was isolated from L4–L6 DRGs and the respective segments of spinal cord using the mirVana miRNA Isolation Kit (Applied Biosystems) according to the manufacturer´s instructions. Afterwards, RNA concentrations were quantified with a NanoDrop ND-1000 spectrophotometer (NanoDrop Technologies) and a cDNA synthesis was performed with 200 ng RNA from DRGs and 400 ng RNA from spinal cord. The reverse transcription was performed with the First Strand cDNA Synthesis Kit (Thermo Fisher Scientific) according to the manufacturer’s recommendations. The quantitative real-time PCR was conducted with QuantStudio™ Design & Analysis Software v 1.4.3 (Thermo Fisher Scientific) in a TaqMan® Gene Expression Assay System (Table 1, Thermo Fisher Scientific) according to the manufacturer´s instructions. The raw data were evaluated using the ΔΔC(T) method, as described previously [29, 47].

**Table 1:** List of used TaqMan® gene expression assays

Target	Gene	Article number	Company
ATF3	Activating transcription factor 3	Mm00476033_m1	Thermo Fisher
cFOS	FBJ osteosarcoma oncogene	Mm00487425_m1	Thermo Fisher
GAPDH	Glyceraldehyde-3-phosphate dehydrogenase	Mm99999915_g1	Thermo Fisher
iNOS	Inducible nitric oxide synthase 2	Mm00440502_m1	Thermo Fisher
MMP9	Matrix metallopeptidase 9	Mm00442991_m1	Thermo Fisher
NFATc3	Nuclear factor of activated T cells, calcineurin dependent 3	Mm01249200_m1	Thermo Fisher
NFATc4	Nuclear factor of activated T cells, calcineurin dependent 4	Mm00452375_m1	Thermo Fisher
NOX2	NADPH oxidase 2	Mm01287743_m1	Thermo Fisher
NOX4	NADPH oxidase 4	Mm00479246_m1	Thermo Fisher
XDH	Xanthine dehydrogenase	Mm00442110_m1	Thermo Fisher

### Multiplex assay

For performing the ProcartaPlex multiplex immunoassay (Thermo Fisher), proteins were isolated with a manufacturer recommended cell lysis buffer, which was further supplemented with a phosphatase inhibitor cocktail (PhosSTOP, Roche) and a protease inhibitor cocktail (cOmplete, Roche). DRG samples were suspended in 100 µl and spinal cord samples in 200 µl of cell lysis buffer. The spinal cord samples were further processed by a cell grinder. Afterwards, the tissue was homogenized two times per sample using a Sonopuls Sonicator (Bandelin) with the setting 6 × 10%. During sonication, the samples were cooled in an ice bath, preventing proteins from degradation. Finally, the samples were centrifuged with 16,000 × g at 4 °C for 10 min, followed by collecting the supernatant for a protein concentration determination via Bradford.

The ProcartaPlex multiplex immunoassay was performed according to the manufacturer’s recommendations. Briefly, a dark wall 96-well plate was prepared by several washing steps and coating steps with respective magnetic beads. Afterwards, standards were prepared in a serial dilution (1:4, (v/v)) and added to the plate, followed by further washing steps. Lastly, the samples were diluted (1:2, (v/v)) and added to the plate, which was sealed and incubated for 40 min with 500 rpm on an orbital shaker at room temperature, overnight at 4 °C and further 50 min with 500 rpm at room temperature. On the next day, the detection antibody mixture was added after a washing step and the plate was further incubated on an orbital shaker for 30 min at room temperature. Again, the plate was washed and streptavidin phycoerythrin was added to the plate and incubated for 30 min as described above. After the last washing step, the plate was prepared with reading buffer, incubated for 5 min with 500 rpm on an orbital shaker, and measured with the Luminex 200 system (Bio-Rad).

### Western blot

For Western blot purposes, the tissue of five animals was pooled and proteins isolated from the respective tissue samples. Therefore, 300 µl of cell lysis buffer, which was described in part 2.5, were added to spinal cord samples and 100 µl to DRG samples. The tissue samples were homogenized as previously described and the protein amount was determined by a Bradford assay.

Afterwards, 30 µg tissue lysate was loaded and separated by SDS-polyacrylamide gel electrophoresis (4% stacking gel, 12% running gel) and transferred on a nitrocellulose membrane with the Trans-Blot®Turbo™ Transfer System (BioRad). For total protein detection, the membrane was blocked with TBST buffer (20 mM Tris, 150 mM NaCl and 0.1% Tween20) containing 5% skimmed milk powder at room temperature for two hours, followed by an incubation overnight with primary antibodies at 4 °C: p65 1:500 (8242S, cell signaling technology), IΚBα 1:500 (4812S, cell signaling technology) and IKKβ 1:250 (2370S, cell signaling technology). For phosphorylated protein detection, the membranes were blocked with TBST buffer containing 5% BSA at room temperature for 2 h, followed by an incubation for at least 48 h with primary antibodies at 4 °C: p-p65 1:500 (3033, cell signaling technology) and p-IΚBα 1:250 (2859S, cell signaling technology). Beta-actin 1:1000 (ab8229, abcam) was used as a loading control. For labeling targets, the fluorescent-labeled secondary antibodies anti-rabbit labeled with IRDye680ED from donkey (Licor), and anti-goat labeled with IRDye800CW from donkey (Licor) were used in 1:5000 dilutions for one hour at room temperature. The antibody detection was performed with an Odyssey CLx device from Licor, followed by a quantification with Image Studio Software.

### Flow cytometry analysis

For creating single-cell suspensions, sciatic nerves were sliced with a tissue scissor and spinal cords were briefly pottered with a tissue grinder at first. Afterwards, all tissue samples (DRGs, sciatic nerve and spinal cord) were incubated in 500 µl Dulbecco’s modified Eagle medium (DMEM, Gibco) supplemented with 3 mg/mL collagenase and 1 µl/mL DNase for 30 min at 37 °C. Then, the enzymatic reaction was stopped by further adding 500 µl DMEM supplemented with 10% FCS. To obtain a single-cell suspension, the samples were filtered through a 70-µm cell strainer and centrifuged at 400 g for 5 min. The supernatant was discarded and the cell pellet washed with 500 µl PBS supplemented with 0.5% BSA, followed by a further centrifugation step. Lastly, the cell pellet was suspended in 500 µl PBS supplemented with 0.5% BSA and 1 mM EDTA to prevent cells from aggregating until and during flow cytometry analysis. The flow cytometry analysis was performed essentially as described previously [37]. Single-cell suspensions were blocked with FcR blocking reagent (Miltenyi Biotec) in 0.5% PBS-BSA for 20 min, stained with fluorochrome-conjugated antibodies (Table 2) and analyzed on a FACSymphony A5 flow cytometer (BD Biosciences). The data were analyzed using FlowJo VX (TreeStar). All antibodies and secondary reagents were titrated to determine optimal concentrations. Comp-Beads (BD Biosciences) were used for single-color compensation to create multicolor compensation matrices. For gating, fluorescence minus one control were used. The instrument calibration was controlled daily using Cytometer Setup and Tracking beads (BD Biosciences). For characterization of immune cell subsets, the antibodies in Table 2 were used.

**Table 2:** List of antibodies used for flow cytometry analysis

Target	Dye	Cell type	Identifier	Source
CD11b	BV605	Monocytes, eosinophils, neutrophils	AB_2737951	Biolegend
CD11c	BV711	AMs	AB_2734778	BD Biosciences
CD19	APC-H7	B cells	AB_1645234	BD Biosciences
CD3	APC-Cy7	T cells	AB_1727461	BD Biosciences
CD326	BV711	Epithelial cells	AB_2738022	BD Biosciences
CD4	BV711	CD4 + T cells	AB_2737973	BD Biosciences
CD45	VioBlue	All leukocytes	AB_2659925	Miltenyi
CD8a	BV650	CD8 + T cells	AB_2563056	Biolegend
CD90	PE	Lymphocytes	AB_2659874	Miltenyi
F4/80	PE-Cy7	Macrophages	AB_893478	Biolegend
GITR	FITC	Tregs	AB_1089125	Biolegend
Ly6C	PerCP-Cy5.5	Monocytes	AB_1727558	BD Biosciences
Ly6G	APC-Cy7	Neutrophils	AB_10640819	Biolegend
MHCII	APC	cDCs, IMs	AB_313329	Miltenyi
NK1.1	BV510	NK cells	AB_2738002	Biosciences
SiglecF	PE	Eosinophils	AB_394341	Biolegend
γδ TCR	APC	γδ T cells	AB_1731813	Biolegend

### Isolation and purification of dorsal root ganglia (DRGs)

For methods using sensory neurons in cell culture, DRGs were dissected and transferred into ice-cold HBSS with CaCl_2_ and MgCl_2_ (Gibco) directly after dissection. Afterwards, DRGs were treated with a collagenase/dispase solution, 500 U/mL collagenase and 2.5 U/mL dispase diluted in neurobasal medium (Gibco), at 37 °C for 75 min. The collagenase/dispase solution was removed by centrifuging and discarding the supernatant, the sensory neurons were washed twice with neurobasal medium containing 10% FCS, followed by an incubation with 0.05% trypsin (v/v) (Gibco) for another 10 min. The washing steps were repeated, and the cells were mechanically dissociated in neurobasal medium (Gibco) supplemented with L-glutamine (2 mM; Gibco), penicillin (100 U/mL; Gibco), streptomycin (100 µg/mL; Gibco), B-27 (Gibco) and gentamicin (50 µg/mL; Gibco). Afterwards, the cell solution was plated on poly-L-lysine-coated cover slips or 48-well plates. After two hours of incubation, 2 mL of neurobasal medium was added and the cells were incubated overnight at 37 °C. For further investigations, the cultured DRGs were further used and treated as described in part 2.9 and 2.13.

### Calcium imaging

For calcium imaging, the cultured sensory neurons were stained with fura-2-AM (Thermo Fisher) for at least 60 min at 37 °C and washed afterwards twice with Ringer´s solution. This was set up freshly with 145 mM NaCl, 1.25 mM CaCl_2_ × 2H_2_O, 1 mM MgCl_2_ × 6 H_2_O, 5 mM KCl, 10 mM D-glucose and 10 mM HEPES and adjusted to a pH of 7.3. During the experiments, Ringer’s solution was also used for baseline measurements and for washing out agonists and compounds between stimulations. For investigating the effect of SAFit2 on different TRP channels, the sensory neurons were pre-incubated with the respective compound for 2 min and stimulated with respective agonists afterwards: 100 nM capsaicin for 30 s (TRPV1 agonist) and 100 mM allyl isothiocyanate for 45 s (TRPA1 agonist). Control experiments were performed with the respective volume of the corresponding vehicle. All stimulating compounds were dissolved in Ringer’s solution to their final concentration. The measurements were performed using a DMI4000 B Microscope, the compact light source CTR550 HS (Leica) and the ValveBank II system (AutoMate Scientific).

### Flexstation method

Cells were grown in 96-well black-walled plates. Human TRPV1 was transiently expressed in HEK-293t cells using jetPEI transfection reagent (Polyplus Transfection). Cells were loaded with Calcium 6 for two hours (Calcium 6 Kit; Molecular devices, San Jose, CA), in an extracellular solution containing 145 mM NaCl, 5 mM KCl, 10 mM glucose, 10 mM HEPES, 1.25 mM CaCl_2_, and 1 mM MgCl_2_, buffered to pH 7.4 with NaOH. According to the manufacturer's protocol, cells are not washed, but extracellular dye is chemically quenched. Calcium 6 fluorescence excited at 488 nm every 2.5 s served as an index of intracellular calcium. Assays were performed at 25 °C. A volume of 50 µL of each applied solution was pipetted automatically according to a preset protocol into 100 µL of extracellular solution in the wells. Fluorescence change was measured by a Flexstation3 (Molecular devices) and is reported relative to baseline fluorescence.

### Transfection of HEK-293 cells

For functional expression of green fluorescent protein (GFP) flanked TRPV1, HEK-293 cells were transfected 16–24 h before the start of patch-clamp experiments using TransfeX™ Transfection Reagent (LGC Standards GmbH) according to the manufacturer’s instructions. HEK-293 cells (German Collection of Microorganisms and cell cultures) were grown at 37 °C in a humidified 95% air/ 5% CO2 incubator in Dulbecco’s modified Eagle medium (DMEM; Gibco) supplemented with 10% v/v heat-inactivated fetal bovine serum, 100 U/mL penicillin G, 100 μg/mL streptomycin sulfate and 2 mM L-glutamine (all from Invitrogen). After reaching approximately 80% confluence HEK-293 cells were transfected in a 35-mm petri dish with 1 µg of hTRPV1_GFP (NM_080705, RG217713, OrgiGene) plasmid DNA.

### Electrophysiology

On the day of experiment, transfected HEK-293 cells were separated by trypsinization, seeded at low density on coverslips (15 mm in diameter), and then incubated for 2 to 4 h to allow adhering of cells on the glass surface. For patch-clamp experiments, coverslips were transferred to a perfusion chamber filled with bath solution and placed on the stage of an inverted microscope. Transfected cells were identified by the fluorescence of the GFP fused to the C-terminus of TRPV1. Patch-clamp experiments were performed in the whole-cell configuration with an EPC-10 patch-clamp amplifier (HEKA Elektronik) at room temperature (20–25 °C). Patch-pipettes were pulled from borosilicate capillaries (DWK Life Sciences) using a single stage glass microelectrode puller (PP-830) resulting in pipettes with 1.5–3 MOhm resistances. The capillaries were coated with Sigmacote® (Merck KGaA) and baked after pulling at 65 °C for 45 min. The pipette solution contained: 140 mM CsCl, 1.93 mM CaCl_2_, 2 mM MgCl_2_, 5 mM 4-(2-hydroxyethyl)-1-piperazineethanesulfonic acid (HEPES), 5 mM ethylene glycol-bis (β-aminoethyl ether)-N,N,N′,N′-tetraacetic acid (EGTA), and 2 mM MgATP. pH and osmolarity were adjusted to 7.2 with 1 M CsOH and 320 mOsmol/kg with D-mannitol, respectively. The bath solution contained: 140 mM NaCl, 5 mM KCl, 2 mM CaCl_2_, 1 mM MgCl_2_, 10 mM HEPES and 10 mM D-glucose. pH and osmolarity were adjusted to 7.4 with 1 M NaOH and 320 mOsmol/kg with D-mannitol, respectively. Capsaicin (M2028, Sigma Aldrich), SAFit2 and Ruthenium Red (RuR) (557,450, Sigma Aldrich) stock solutions were prepared by dissolving 10 mM, 2.5 mM and 10 mM in DMSO, respectively. Capsaicin, SAFit2 and RuR supplemented bath solutions were prepared directly before the measurements by adding the appropriate volume of stock solutions to the external solution. Bath solution exchange was performed using a gravity flow perfusion system. Whole-cell currents were elicited by repetitively applying 500 ms voltage pulses from − 120 mV to + 80 mV (20 mV increments) from a holding potential of − 40 mV and 500 ms voltage ramps from − 60 mV to + 60 mV from a holding potential of − 60 mV. Currents were recorded with a 10 kHz low-pass filter and sampled with a frequency of 50 kHz. Data were memorized with Patchmaster (HEKA Elektronik) and analyzed with Fitmaster (HEKA Elektronik). To account for the different sizes of the measured cells, whole-cell currents were normalized to the cell membrane capacity.

### Calcitonin gene-related peptide assay

For measuring the effect of SAFit2 on calcitonin gene-related peptide (CGRP) secretion, DRGs were isolated as previously described (part 2.8) and stimulated with the respective compound the next day. Therefore, the cells were washed with HBSS and incubated with 250 µl of the respective compound, diluted in HBSS to their final concentration, and 1 µM capsaicin for 15 min at 37 °C. The respective volume of the vehicle was used as a negative control, and 1 µM capsaicin alone as positive control. The CGRP quantification was performed with an ELISA kit from Bertin Bioreagent, which was used according to the manufacturer´s recommendations. The final detection was performed measuring the absorption at 405 nm. The raw data were evaluated using linear regression according to the manufacturer´s recommendation.

### Calcineurin assay

The calcineurin activation was measured with a cellular calcineurin phosphatase activity assay kit from abcam, which was used according to the manufacturer’s instructions. The enzyme calcineurin was isolated from HEK-293 cells using 1 mL of TBS buffer containing 20 mM Tris and 150 mM NaCl (adjusted to pH 7.3) for a 90% confluent T75 flask. The cell lysate was further purified according to the kit protocol. To measure the impact of the respective compounds on the activity of calcineurin, the purified cellular extract with calcineurin was supplemented with the respective compound and added to the prepared assay plate. Afterwards, the plate was incubated for 30 min at 30 °C to stimulate the activity of the enzyme for a distinct period of time. Finally, the reaction was terminated by adding the assay reagent to the plate and the absorption was detected after 20 to 30 min at 620 nm. The raw data were evaluated using linear regression according to the manufacturer’s instructions.

### Differentiation of bone marrow-derived macrophages (BMDM)

For bone marrow isolation, mice were killed and hind legs were dissected and transferred into ice-cold PBS. The bones were cleaned from muscle tissue, cut open and transferred into 0.5-mL Eppendorf tubes with holes at their peak end. To isolate the bone marrow via centrifugation (1300 rpm, 1 min, RT), the tubes were placed into 1.5-mL Eppendorf tubes, containing 50 µL of BMDM medium (RPMI + GlutaMAX (Thermo Fisher Scientific) supplemented with 10% FCS (Sigma-Aldrich), 100 U/mL penicillin + 100 µg/mL streptomycin (Thermo Fisher Scientific) and 20 ng/mL M-CSF (Peprotech). After centrifugation, the bone marrow pellet was resuspended in BMDM medium and distributed equally into a 6-well plate. For macrophage differentiation, cells were incubated overnight at 37 °C, followed by a change of BMDM medium the next day. At day four of differentiation, fresh growth factors were provided by adding the same amount of BMDM medium to the differentiating cells. After 7 days, the cells were fully differentiated and used for further experiments [57].

### Human macrophage isolation and purification

For isolating buffy coats from whole bloods samples, 50-mL Leucosep tubes were equilibrated with 15 mL lymphocyte separation medium, which was passed through the membrane by centrifuging at 1000 × *g* for 5 min. Afterwards, 40 mL whole blood was transferred into a tube, which was then filled up to 50 mL with PBS containing 2 mM EDTA. The samples were centrifuged at 440 × *g* for 35 min at room temperature without break to enable a clear separation of fractions comprising a plasma fraction at the top, the buffy coat in the middle and an erythrocyte and granulocyte fraction at the bottom. The plasma was discarded and the buffy coat transferred into a fresh 50-mL tube, which was then again filled up to 50 mL with PBS containing 2 mM EDTA for purification. The suspension was again centrifuged at 500 × *g* at room temperature for 5 min and the purification step was repeated until the supernatant was almost clear. Afterwards, the cell pellet was resuspended in 50 mL RPMI medium supplemented with 1% P/S and the cell solution was plated with 1 mL/ well into 6-well plates. The cells were incubated for one hour at 37 °C, followed by a medium change with 2 mL RPMI supplemented with 1% P/S and 3.3% human plasma for differentiation purpose. The medium was changes every 2–3 days until day seven of differentiation. Monocytes were now fully differentiated into macrophages and can be used for further experiments [43].

### Transwell assay

For investigating the migration of macrophages, cultivated macrophages were starved overnight and harvested the next day by incubating them 10 min with 600 µl accutase/ well (Sigma-Aldrich) at 37 °C and using a cell scraper. The reaction was terminated using 1% (w/v) BSA solution for murine macrophages. Afterwards, the macrophages were centrifuged at 1000 rpm for 5 min and the cell pellet was resuspended in 1 mL of the respective starving medium. The cell concentration was determined, using a Neubauer counting chamber and the cell concentration adjusted to 10^6^ cells/mL. Transwell inserts (Greiner) were placed into 24-well plates, which contain 500 µl medium/well with either 10% FCS as chemoattractant for murine cells or 3.3% human plasma for human cells and the respective compound. The cells were seeded by adding 300 µl cell solution per insert. After incubating the setup for two hours at 37 °C, the inserts were rinsed with PBS and cells were fixed with 2% PFA for two minutes, followed by further washing steps and a further fixation and permeabilization step with 100% ice-cooled methanol for 20 min. Lastly, the membranes were stained with DAPI in a final concentration of 0.2 ng/mL in the dark for two minutes and embedded on slides with mounting media for quantification purposes. Five images were taken per membrane with the fluorescence microscope Observer.Z1 (Carl Zeiss) and quantified with ImageJ software.

### Cytotoxicity assay with WST-1

Primary murine macrophages were isolated and differentiated as previously described (part 2.15). The cells were harvested the same way as described in part 2.17. Afterwards, the cell concentration was adjusted to 3.6 × 10^5^ cells/ mL and cells were seeded with 1 mL/ well into 24-well plates, followed by an incubation for further 48 h at 37 °C. For analyzing the compound´s cytotoxicity, DMEM medium was supplemented with 10% (v/v) WST-1 and either the compound of interest or the according volume of the dissolvent. The cells were treated with 300 µl of prepared medium for two hours at 37 °C and the absorption wavelength was measured at 450 nm and 600 nm afterwards.

### Seahorse bioanalyzer

For measuring the metabolic parameters with the Seahorse bioanalyzer device, primary murine macrophages were isolated and differentiated as previously described (part 2.15). The macrophages were harvested in the same way as described in the part 2.17, seeded (25 × 10^3^ cells/well) into Seahorse 96-well cell culture plates and incubated overnight. On the day of measurement, the media was replaced by XF RPMI medium supplemented with 10 mM glucose and 2 mM glutamine (all from Agilent). The plate was equilibrated for 30 min in a non-CO_2_ incubator at 37 °C. Metabolic parameters were measured on a Seahorse XFe 96 extracellular flux analyzer (Agilent). To analyze the ATP production rate, 2.5 µM oligomycin and 500 nM rotenone together with antimycin A were sequentially added to the cells. All chemicals were purchased from Cayman chemicals. Data were processed using Wave Desktop (Version 2.6.0.31) and ATP rates were calculated using the Seahorse Analytics online tool (Version from February 2022).

### Data analysis and statistics

All data are presented as mean ± SEM. Normal distribution was confirmed using the Shapiro–Wilk test. For in vitro experiments comparing only two groups, an unpaired and heteroskedastic Student’s *t* test was conducted with Welch’s correction. When comparing more than two groups, a one-way analysis of variance (ANOVA) was used and for the comparison of more than three groups a two-way ANOVA was conducted. For statistical analysis of behavioral experiments, an ANOVA was performed, followed by Bonferroni’s post hoc correction. For all statistical analysis the software GraphPad Prism 9 was used. A *p* value of < 0.05 was considered statistically significant.

## Results

### SAFit2 ameliorates nerve injury-induced mechanical hypersensitivity

Our initial goal was to investigate whether SAFit2 has an influence on nerve injury-induced neuropathic pain. Therefore, we chose the robust and well described spared nerve injury (SNI) model to generate neuropathic pain in mice [9] and treated the mice intraperitoneally either with 10 mg/kg SAFit2 or vehicle from day five to ten after the surgery.

**Fig. 1:**
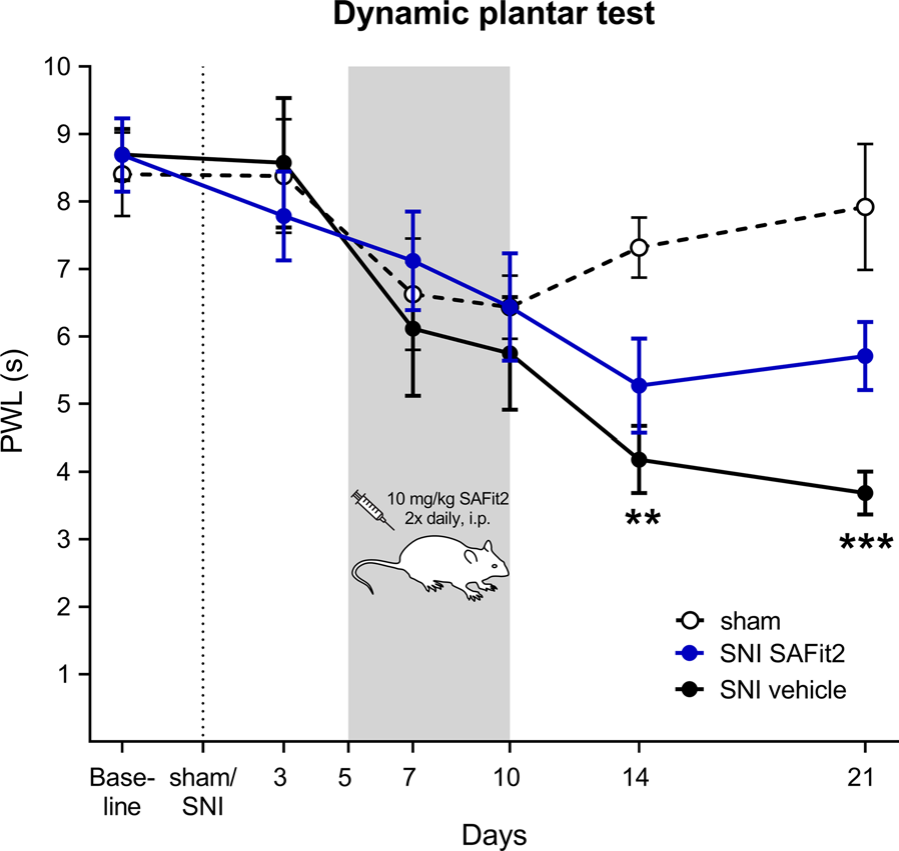
Reduction of nerve injury-induced mechanical hypersensitivity in mice after SAFit2 treatment. Baseline levels were measured one day before the surgery. During the surgery, two of the sciatic nerve branches were dissected to induce neuropathic pain. Afterwards, the mice were treated intraperitoneally with 10 mg/kg SAFit2 from day five to ten to analyze the impact of SAFit2 on neuropathic pain behavior. The mechanical pain threshold was assessed over period of 21 days as indicated. The data represent the mean ± SEM from 13–14 mice per group. **p < 0.01, ***p < 0.001, two-way ANOVA with Bonferroni´s post hoc test. *PWL* paw withdrawal latency, *SNI* spared nerve injury, *SAFit2* selective antagonist of FKBP51 by induced fit 2

To analyze neuropathic pain, we determined the mechanical pain threshold of mice paws in a dynamic plantar test over 21 days (Fig. 1). After the surgery, the vehicle-treated SNI animals developed a significant mechanical hypersensitivity over time as compared to the sham-treated animals. In contrast, the SAFit2-treated SNI animals showed less mechanical hypersensitivity than the vehicle-treated animals (Fig. 1).

### SAFit2 reduces cytokine and chemokine levels in dorsal root ganglia and spinal cord after nerve injury

**Fig. 2:**
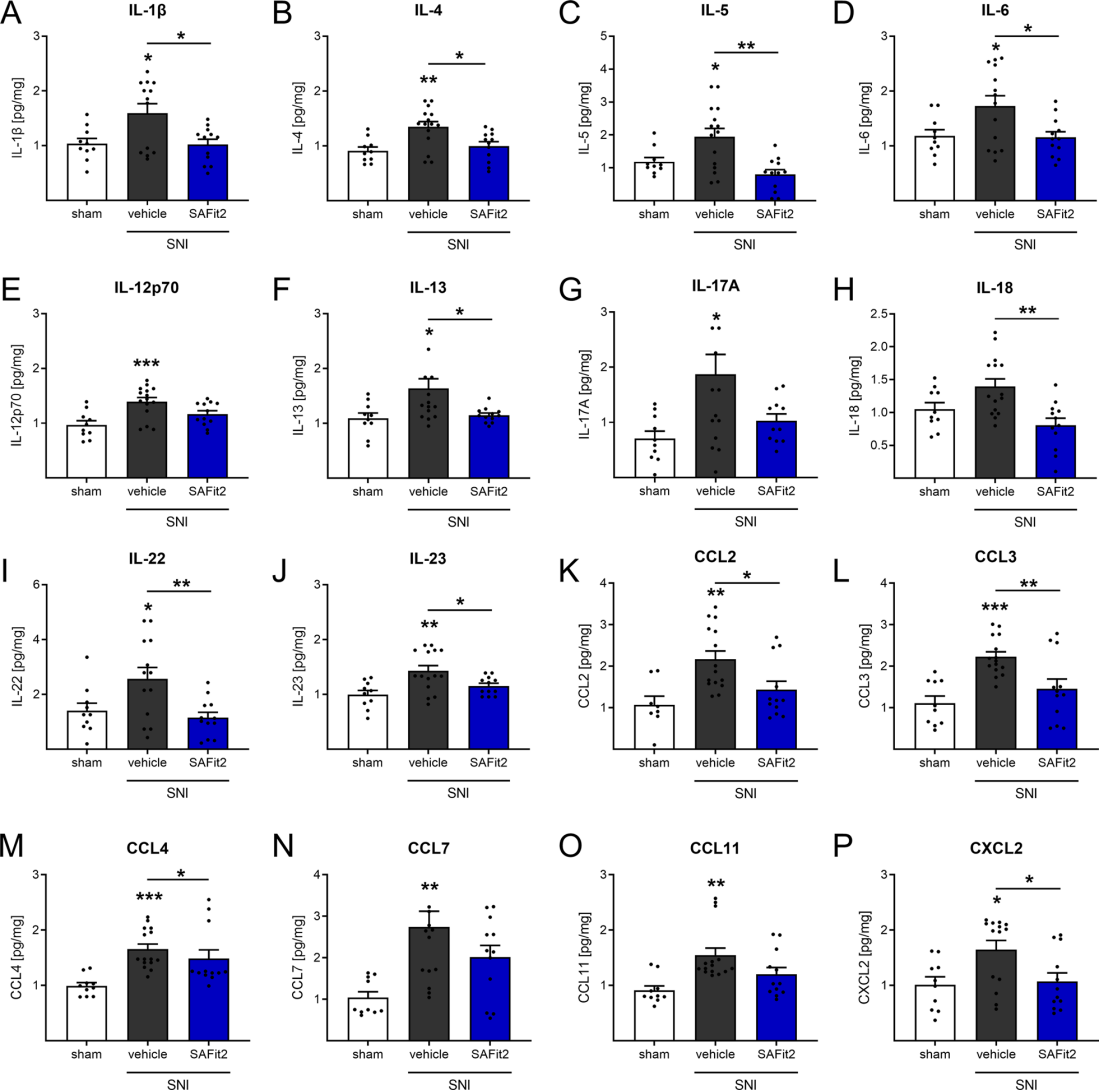
Reduced cytokine and chemokine levels in lumbar DRGs of SAFit2-treated SNI mice after 21 days. Mice underwent SNI surgery and were then treated with either vehicle or 10 mg/kg SAFit2 from day five to ten after the surgery. After 21 days, L4–L6 DRGs from ipsilateral and contralateral sides were isolated, lysed and tested in a multiplex immunoassay of 26 cytokines and chemokines. Shown is a section of significantly altered interleukins (**A**–**G)** and chemokines (**K**–**P)** after SAFit2 treatment. The data represent the mean ± SEM from 5 mice per group, measured in technical triplicates, respectively. The raw data were related to the total protein amount of the sample and the ipsilateral (injured) value was normalized to the contralateral (control) value per animal. *p < 0.05, **p < 0.01, ***p < 0.001 one-way ANOVA with Tukey´s post hoc test with multiple comparisons, comparing all treatments, respectively. *SNI* spared nerve injury, *DRGs* dorsal root ganglia, *SAFit2* selective antagonist of FKBP51 by induced fit 2

**Fig. 3:**
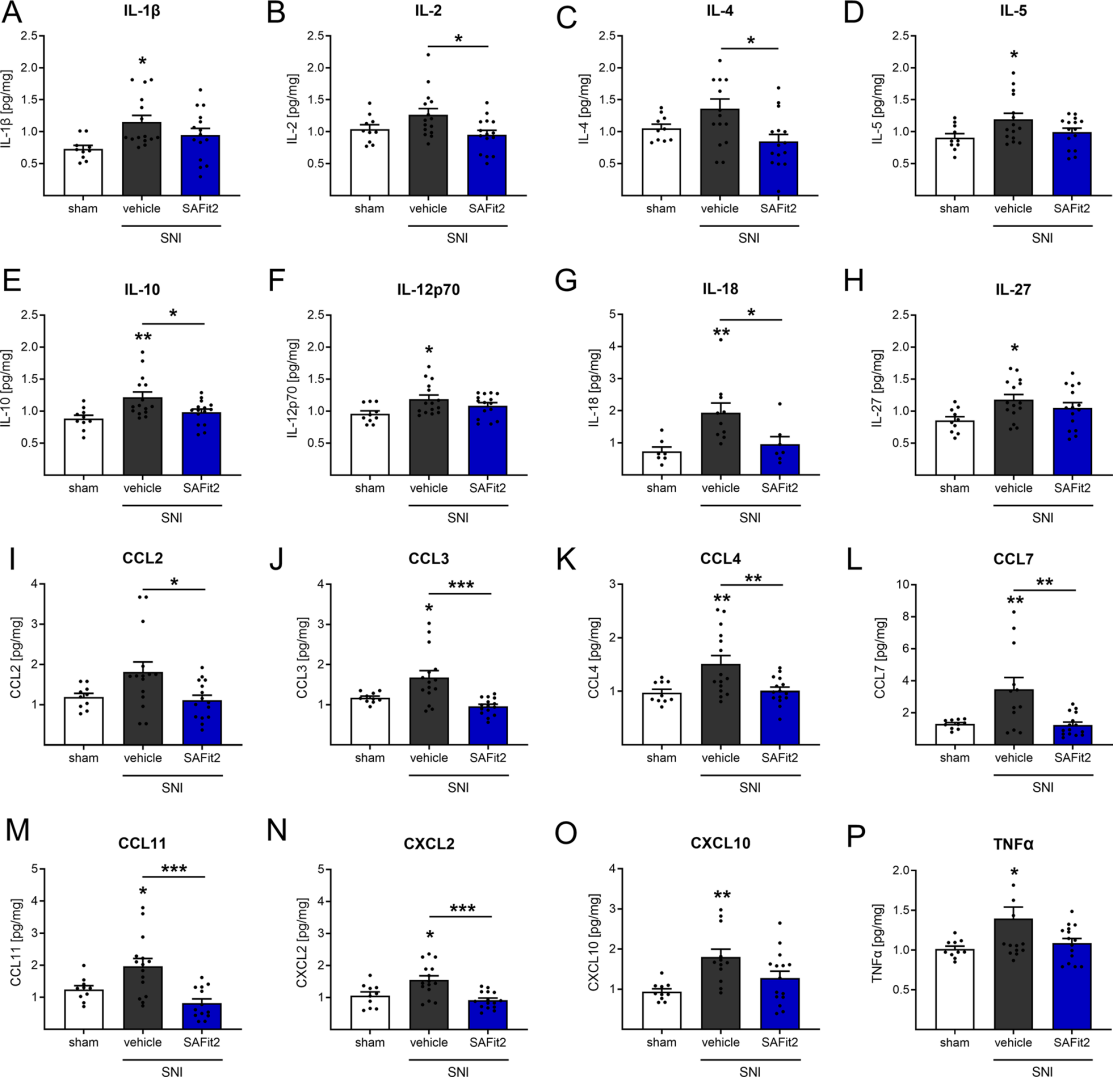
Reduced cytokine and chemokine levels in spinal cord of SAFit2-treated SNI mice after 21 days. Mice underwent SNI surgery and were treated with either vehicle or 10 mg/kg SAFit2 from day five to ten after the surgery. After 21 days, the spinal cord from ipsilateral and contralateral sides was isolated, lysed and analyzed using a multiplex immunoassay including a panel of 26 cytokines and chemokines. This figure displays a section of significantly altered cytokines (**A**–**H**, **P)** and chemokines (**I**–**O)** after SAFit2 treatment. The data represent the mean ± SEM from 5 mice per group, measured in technical triplicates, respectively. The raw data were related to the total protein amount of the sample and the ipsilateral (injured) value was normalized to the contralateral (control) value per animal. *p < 0.05, **p < 0.01, ***p < 0.001 one-way ANOVA with Tukey´s post hoc test with multiple comparisons, comparing all treatments, respectively. *SNI* spared nerve injury, *SAFit2* selective antagonist of FKBP51 by induced fit 2

Since we detected that SAFit2 reduces the mechanical hypersensitivity in nerve-injured mice, we isolated lumbar dorsal root ganglia (DRGs) and spinal cord (SC) at day 21 after the surgery to investigate how amelioration of mechanical hypersensitivity is mediated. Therefore, we analyzed at first the expression of neuronal stress markers (activating transcription factor 3, matrix metallopeptidase 9, cFOS) and oxidative stress markers (xanthine dehydrogenase, NADPH oxidase 2 and 4, inducible nitric oxide synthase) but did not detect any significant differences between vehicle and SAFit2-treated animals (Additional file 1: Fig. S1). Since we did not observe any changes in the expression of common stress markers, we next analyzed the concentrations of inflammatory and proalgesic mediators in DRGs and spinal cord. We therefore performed a multiplex immunoassay to detect alterations in inflammatory and pain-mediating cytokines and chemokines after SAFit2 treatment (Figs. 2, 3, Additional file 1: Fig. S2).

All cytokines and chemokines were significantly upregulated in lumbar DRGs of vehicle-treated SNI animals (Fig. 2A–G, I–P) as compared to sham-treated animals, except for interleukin (IL)-18 (Fig. 2H). However, DRGs of SAFit2-treated SNI mice showed quite similar cytokine and chemokine levels as the DRGs of sham-treated mice (Fig. 2A–P). Moreover, we detected significant differences between vehicle and SAFit2-treated mice for both pro-inflammatory cytokines as IL-1β, IL-6, IL-18, IL-23 (Fig. 2 A, D, H, J) and anti-inflammatory cytokines as IL-4, IL-5 IL-13, IL-22 (Fig. 2B, C, F, I). Also, significant differences were observed for pain-mediating chemokines as C-C motif ligand (CCL) 2, CCL3, CCL4 and C-X-C ligand 2 (Fig. 2K, L, M, P). In DRGs SAFit2 overall reduced cytokine and chemokine levels after nerve injury, which were thereby largely similar to levels in sham operated animals.

We next analyzed the concentrations of cytokines and chemokines in the dorsal spinal cord (Fig. 3). Interestingly, the analysis of spinal cord samples revealed similar trends for SAFit2 treatment as shown in the DRG measurements, including an increase of mediators after vehicle treatment which was blunted after SAFit2 treatment. However, SAFit2 reduced pain-mediating chemokines CCL2, CCL3, CCL4, CCL7, CCL11 and C-X-C ligand 2 (Fig. 3I–N) more substantially in the spinal cord than in DRG samples. In addition, we observed significant differences in the cytokines IL-2, IL-4, IL-10 and IL-18 (Fig. 3B, C, E, G) for vehicle and SAFit2-treated animals. Overall, SAFit2 treatment had a major effect on chemokine levels in the spinal cord and on cytokine levels in DRGs. In summary, these results indicate that SAFit2 significantly reduces inflammation and pain-mediating cytokine and chemokine secretion after nerve injury.

### SAFit2 in vivo treatment reduces NF-κB pathway activation after SNI

Based on the significant reduction of cytokines and chemokines in lumbar DRGs and spinal cord after SAFit2 treatment, we hypothesized that SAFit2 has an impact on the NF-κB signaling pathway and its activation, since NF-κB is one of the most relevant transcription factors being involved in the regulation of cytokine and chemokine expression. Moreover, FKBP51 has repeatedly been implicated in the NF-κB signaling pathway [17].

To investigate this hypothesis, we again performed an SNI mouse model and treated mice with either vehicle or 10 mg/kg SAFit2 (i.p.) over the same period of time as in the previous experiment. For examining the underlying mechanisms, we isolated lumbar DRGs and the respective parts of the spinal cord at day 14 after SNI surgery. This timepoint represents a phase of acute to chronic neuropathic pain with an established neuroinflammation [9, 44], being appropriate to analyze the ongoing inflammation on its peak.

**Fig. 4:**
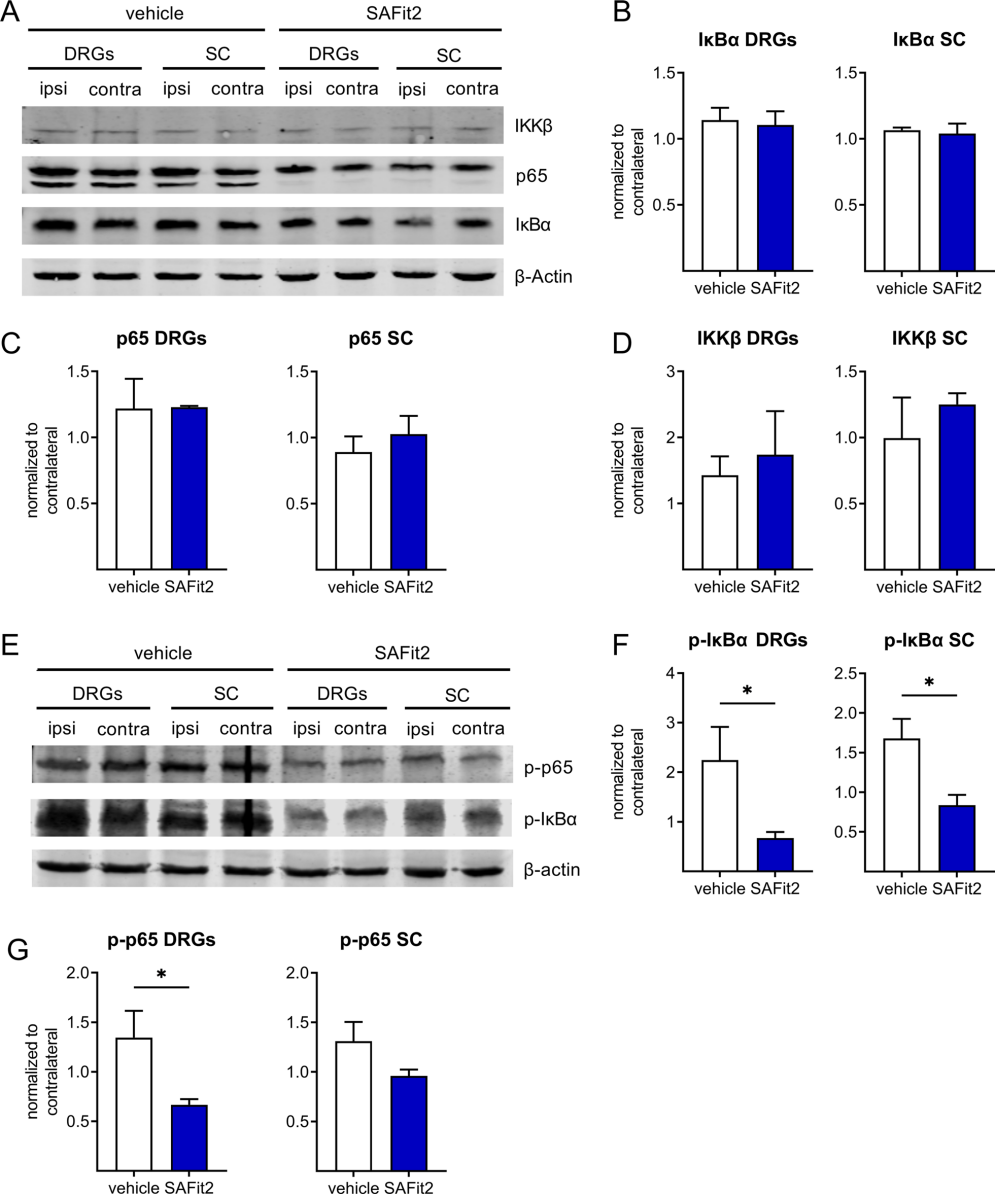
SAFit2 treatment reduces NF-κB pathway activation in SNI-treated mice after 14 days. Mice underwent SNI surgery and were then treated with either vehicle or 10 mg/kg SAFit2 from day five to ten after the surgery. Lumbar L4–L6 DRGs and spinal cord were collected from ipsilateral and contralateral sides at day 14. **A** Representative Western blot of the total protein expression of the factors IKKβ, p65 and IκBα after vehicle and SAFit2 treatment. **B**–**D** Quantification of the total protein expression of IκBα (**B**), p65 (**C**) and IKKβ (**D**). **E** Representative Western blot of phosphorylated IκBα and p65 after vehicle and SAFit2 treatment. **F**, **G** Quantification of phosphorylated IκBα (**F)** and phosphorylated p65 (**G)** levels in DRGs and spinal cord. The data represent the mean ± SEM of 3–5 Western blots measured with the tissue pooled from 5 mice per group. *p < 0.05 Student´s t-test with Welch´s correction. *SNI* spared nerve injury, *DRGs* dorsal root ganglia, *SC* spinal cord, *SAFit2* selective antagonist of FKBP51 by induced fit 2, *IκBα* NF-κB inhibitor alpha, *IKKβ* IκB kinase beta

We performed Western blots with respective tissue lysates, in which we analyzed the total and the phosphorylated amount of three crucial protein members of the NF-κB signaling pathway: the inhibitor complex alpha (IκBα), p65 itself and the upstream IκBα kinase beta (IKKβ). These factors were analyzed in both DRGs and spinal cord, respectively (Fig. 4), observing that SAFit2 treatment has no influence on the total protein amount of IκBα (Fig. 4B), p65 (Fig. 4C) and IKKβ (Fig. 4D).

However, SAFit2 significantly affects the phosphorylation state of the respective factors (Fig. 4E–G). We observed that SAFit2 treatment leads to a significant decrease of IκBα phosphorylation (Fig. 4F) and p65 phosphorylation (Fig. 4G) in lumbar DRGs and spinal cord of nerve-injured mice. Based on this, we concluded that SAFit2 does not change the basal expression of the respective factors, but strongly reduces the NF-κB pathway activation. Besides the NF-κB pathway, the transcription factor NFAT might also have an influence on cytokine and chemokine regulation. However, we detected no alterations in the regulation of NFAT after SAFit2 treatment (Additional file 1: Fig. S1), indicating the NF-κB pathway as primary pathway for cytokine and chemokine production after nerve injury.

### SAFit2 treatment reduces immune cell infiltration into dorsal root ganglia and spinal cord after nerve injury

**Fig. 5:**
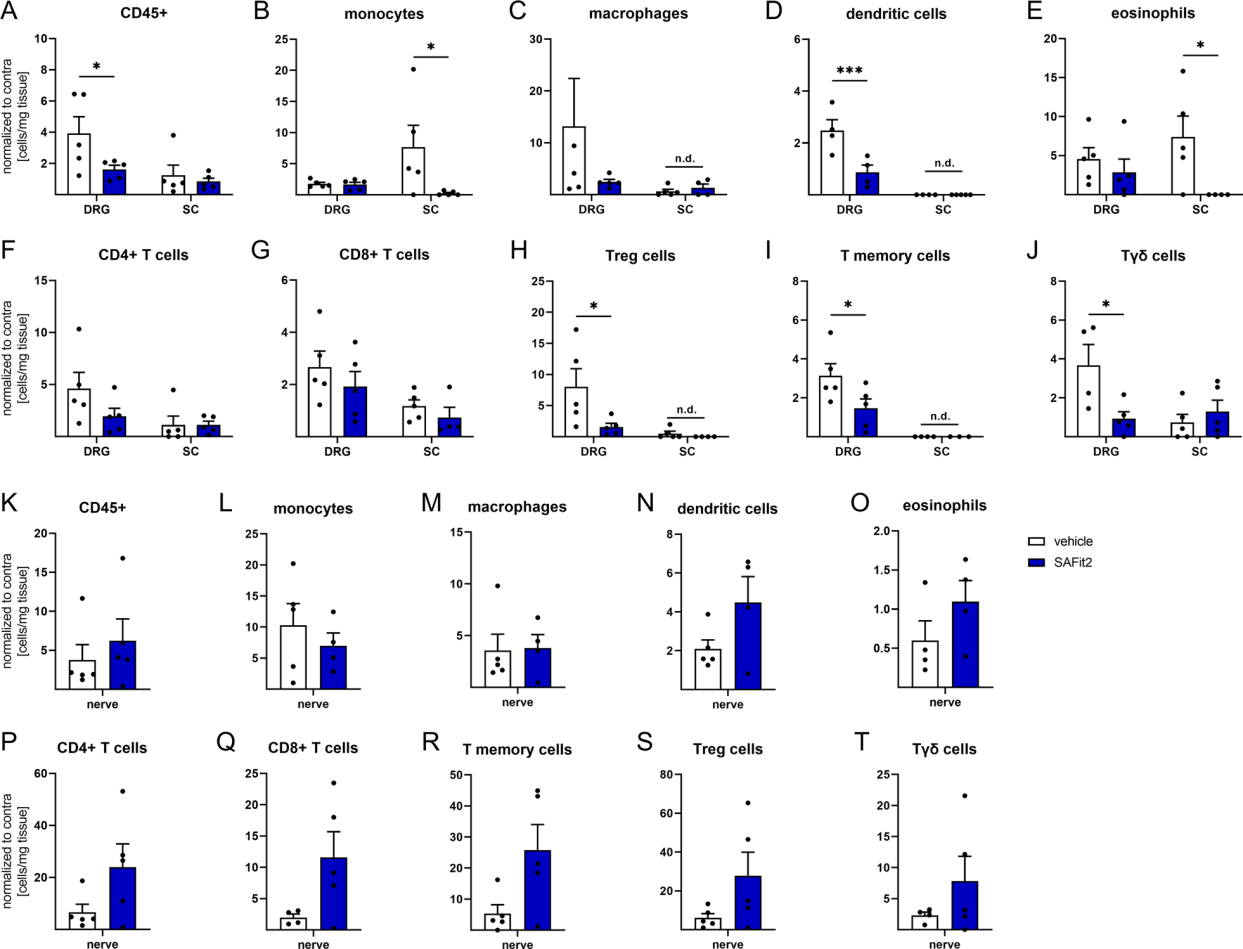
Immune cell reduction in DRGs and spinal cord of SAFit2-treated mice 14 days after SNI. Mice underwent SNI surgery and were then treated with either vehicle or 10 mg/kg SAFit2 from day five to ten after the surgery. L4–L6 DRGs, spinal cord and sciatic nerve were collected from ipsilateral and contralateral sides at day 14 after the surgery. **A** Flow cytometry analysis was performed with tissue lysates to analyze the immune cell distribution after SAFit2 treatment. **A**–**J** Relative immune cell amount in L4–L6 DRGs and spinal cord. **K**–**T** Relative immune cell amount in sciatic nerves. The data represent the mean ± SEM from 5 mice per group. *p < 0.05, ***p < 0.001 two-way ANOVA with Sidak´s post hoc test for DRGs and SC figures, *p < 0.05 Student´s *t*-test with Welch´s correction for sciatic nerve figures. *SNI* spared nerve injury, *DRGs* dorsal root ganglia, *SC* spinal cord, *SAFit2* selective antagonist of FKBP51 by induced fit 2, *n.d.* not detected

Since modulation of the NF-κB signaling pathway activation can influence immune cell migration [19, 28, 39], we next investigated the influence of SAFit2 on the immune cell distribution in neuronal tissue after nerve injury. We hypothesized that a reduced immune cell infiltration would additionally explain reduced cytokine and chemokine levels. Therefore, we performed the same in vivo experiment, an SNI surgery followed by SAFit2 treatment, and collected lumbar DRGs, the respective parts of the spinal cord and both sciatic nerves 14 days after surgery. The tissue was gently homogenized, and the subset of immune cells determined in the respective tissues using a flow cytometry analysis (Fig. 5).

Interestingly, we detected a significant reduction of all immune cells in the DRGs (CD45 + , Fig. 5A) which was not the case for the inflamed sciatic nerve which is the core of inflammation (Fig. 5K). Moreover, we observed a significant reduction of dendritic cells (Fig. 5D), regulatory T cells (Fig. 5H), memory T cells (Fig. 5I) and gamma delta T cells (Fig. 5J) in the DRGs after SAFit2 treatment, which we could not observe in the sciatic nerve samples. Instead, a slight upregulation of the respective immune cells was measured in the sciatic nerve samples (Fig. 5N, R, S, T). A significant decrease of monocytes (Fig. 5B) and eosinophils (Fig. 5E) was detected in the spinal cord after SAFit2 treatment. In contrast, these immune cell types were unaltered in the sciatic nerve samples comparing vehicle and SAFit2-treated animals (Fig. 5L and O). Also, the total amount of immune cells was not altered by SAFit2 treatment in the sciatic nerve samples (CD45 + , Fig. 5K). In summary, SAFit2 treatment in vivo leads to a reduced infiltration of immune cells into DRGs and spinal cord, but it does not affect the number nor the distribution of immune cells at the sciatic nerve.

### SAFit2 reduces the migration of primary murine and human macrophages

As we have seen significant changes in the number of immune cells in neuronal tissue after SAFit2 treatment and the counteracting effect of the compound on the pro-migratory NF-κB pathway, we next assessed whether SAFit2 has a direct effect on the migratory behavior of antigen presenting cells. In particular, we focused on analyzing the impact of SAFit2 on macrophage migration since macrophages constitute an essential role in neuroinflammation and central sensitization [62]. In addition, the target FKBP51 was previously suggested to be involved in promoting cell migration [32, 54].

**Fig. 6:**
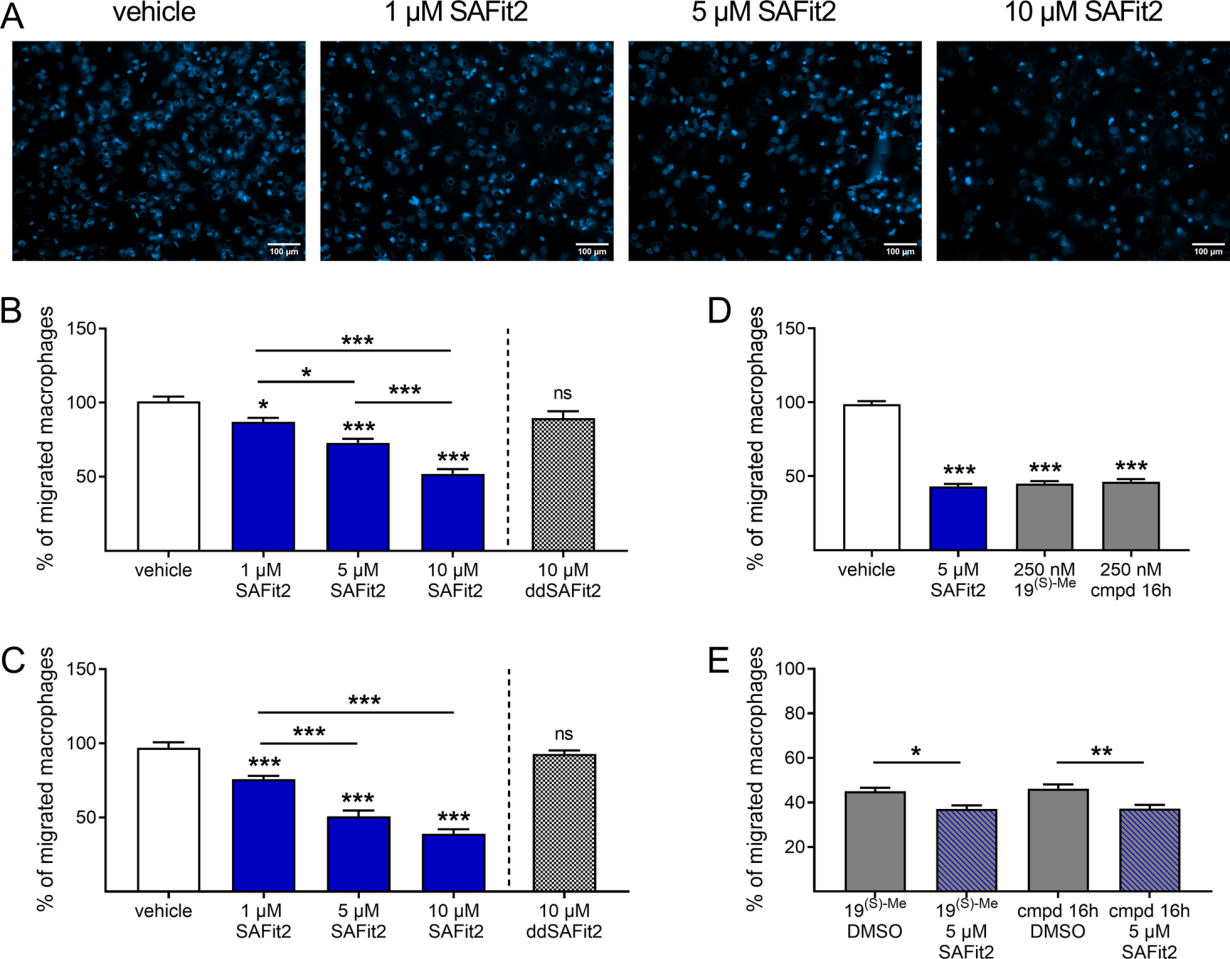
SAFit2 reduces murine and human macrophage migration in vitro. Primary monocytes were isolated and differentiated into macrophages in vitro. The effect of SAFit2 on macrophage migration was assessed in Transwell assays in which the cells were treated either with different concentrations of SAFit2 or with ddSAFit2 for two hours. Other structurally different FKBP51 inhibitors (19^(S)−Me^ [26] and cmpd 16 h [41]) were added already 24 h previous the Transwell assays and were kept in medium during the assay. **A** Representative images of stained membranes displaying reduced murine macrophage migration after SAFit2 treatment. **B** Quantification of murine macrophage migration after SAFit2 and ddSAFit2 treatment. **C** Quantification of human macrophage migration after SAFit2 and ddSAFit2 treatment. **D** Quantification of murine macrophage migration comparing SAFit2 treatment with other unspecific FKBP51 inhibitors. **E** Quantification of murine macrophage migration comparing unspecific FKBP51 inhibitor treatment with or without additional SAFit2 treatment. The data represent the mean ± SEM of 45–75 quantified images from 9 to 15 membranes per group, which were measured in three runs from 3 to 6 different biological replicates. *p < 0.05, **p < 0.01, ***p < 0.001 one-way ANOVA with Tukey´s post hoc test. *cmpd* compound, *SAFit2* selective antagonist of FKBP51 by induced fit 2

For investigating the migration of primary macrophages, we isolated monocytes either from murine bone marrow or from human blood donations and differentiated the monocytes into primary macrophages in cell culture. Afterwards, their migratory behavior was assessed in Transwell assays during the treatment with SAFit2 (Fig. 6). Interestingly, SAFit2 significantly reduced the migration of both murine (Fig. 6B) and human macrophages (Fig. 6C) in a concentration dependent manner. However, ddSAFit2 (Additional file 1: Fig. S3), which corresponds almost to the structure of SAFit2 as chiral analogue and is instead biologically inactive, does not affect the migration of murine and human macrophages (Fig. 6B, C). As a control experiment, we analyzed the impact of SAFit2 on murine macrophage viability and metabolism to exclude any cytotoxic effect which could also reduce the total amount of migrated macrophages (Additional file 1: Fig. S4). However, SAFit2 has neither a detectable influence on cell viability nor on oxidative phosphorylation, adenosine triphosphate (ATP) synthesis and mitochondrial oxygen consumption rate (Additional file 1: Fig. S4).

As we discovered that SAFit2 reduces macrophage migration, we wanted to further investigate whether this effect is on target (i.e., mediate by FKBP51) or a potential off-target effect of the compound (Fig. 6D, E). Therefore, we again performed Transwell assays with primary murine macrophages, however pretreating the cells this time with two FKBP inhibitors from a different chemical series [26, 41]. The concentration and treatment duration for a successful inhibition were chosen based on previous intracellular FKBP51 occupancy studies [16]. These FKBP inhibitors also reduced the murine macrophage migration at low concentrations (Fig. 6D) and in a non-additive manner to SAFit2 (Fig. 6E), strongly suggesting an FKBP as relevant target for the reduction of macrophage migration. Likewise, SAFit2 shows similar effects in low micromolar concentrations after two hours of treatment.

### SAFit2 causes a calcineurin-dependent desensitization of the TRPV1 channel in sensory neurons which reduces the secretion of CGRP

We observed a beneficial role of SAFit2 in nerve injury-induced neuroinflammation. Neuroinflammation is on the one hand mediated by immune cells, but also on the other by an increased activity of sensory neurons and the subsequent release of pro-inflammatory mediators [6, 7]. We therefore hypothesized that SAFit2 may also have an effect on the activity of sensory neurons and subsequently on the downstream signaling that may involve processes which initiate neuroinflammation. More specifically, sensory neurons express ion channels such as the transient receptor potential cation channel subfamily V member 1 (TRPV1) which plays an important role in the development of neuropathic pain. States of hypersensitivity, as in the SNI experiments, can be associated with TRPV1 modulation. To test whether SAFit2 alters the activity of TRPV1, we dissected DRGs from naïve mice and measured the TRPV1 activity via calcium imaging.

**Fig. 7:**
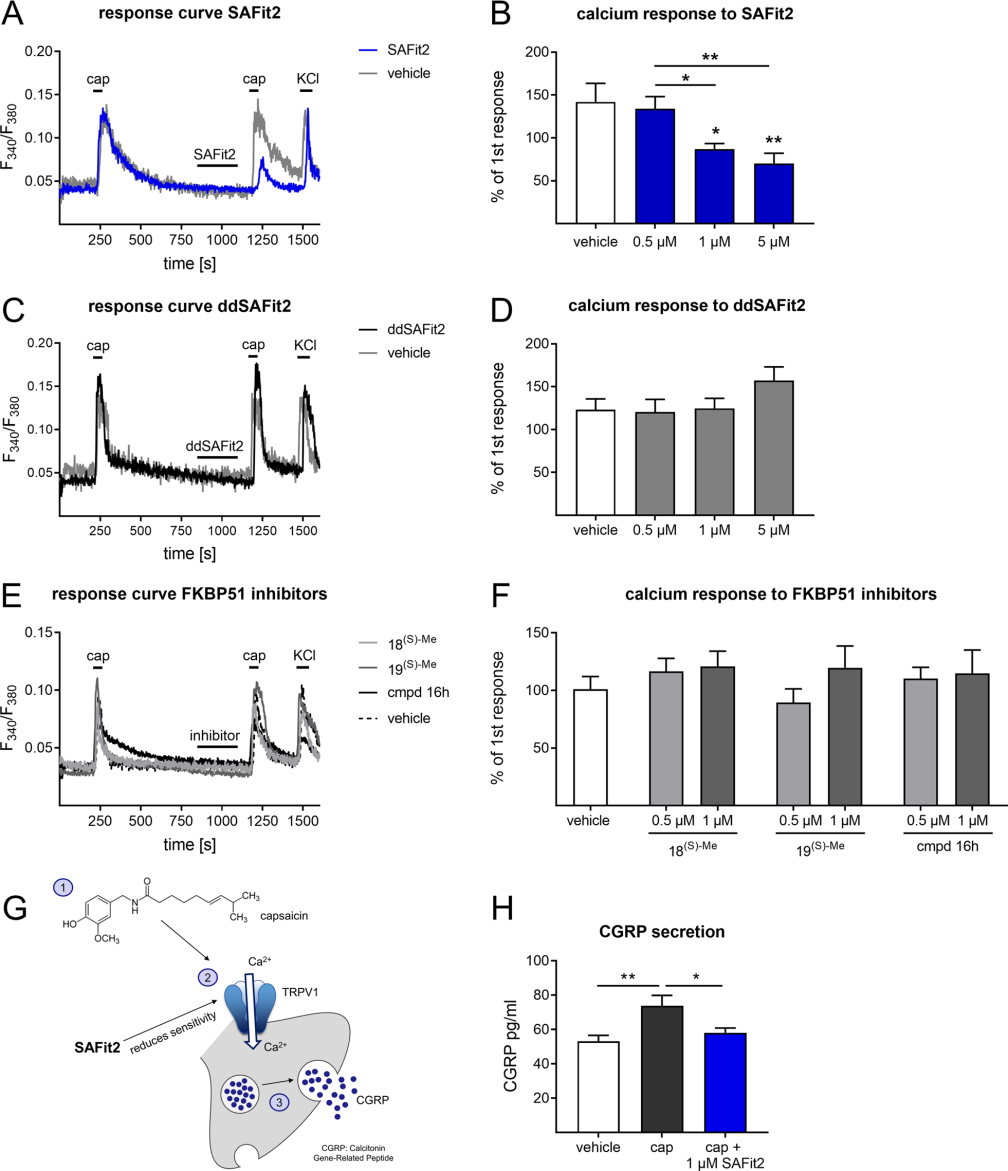
SAFit2 desensitizes the TRPV1 channel and reduces the CGRP secretion in primary sensory neurons. Primary sensory neurons were isolated from mice and the effect of SAFit2 on the TRPV1 channel activity was assessed in calcium imaging experiments. **A**, **C**, **E** Representative traces of calcium influxes in sensory neurons, which were pre-incubated with 5 µM SAFit2 (**A**), 1 µM ddSAFit2 (**C)** or 1 µM structurally unrelated FKBP inhibitors (**E)** for 2 min and stimulated with capsaicin (100 nM, 30 s) afterwards and KCl (50 mM) as a positive control for neuronal response at the end of each experiment. **B**, **D**, **F** Quantification of the treated calcium response related to the untreated calcium response. **G** Schematic illustration of the release of CGRP from sensory neurons after capsaicin treatment. The scheme was created with images from motifolio. **H** Quantification of the amount of CGRP in the supernatant of treated DRG cultures. **B**, **D**, **F** The data represent the mean ± SEM of 25–49 sensory neurons per group. **H** The data represent the mean ± SEM of *n* = 4–5 per group *p < 0.05, ** *p* 0.01, one-way ANOVA with Tukey´s post hoc test. *TRPV1* transient receptor potential cation channel subfamily V member 1, *CGRP* calcitonin gene-related peptide, SAFit2: selective antagonist of FKBP51 by induced fit 2, *cap* capsaicin, *KCl* potassium chloride

For examining the impact of SAFit2 on TRPV1-mediated calcium flux, we established a protocol in which we stimulated TRPV1 twice: at first with capsaicin alone and second after a preincubation with SAFit2 to determine whether SAFit2 alters the channel-mediated calcium influx. The effect of the compound was verified by comparing the calcium fluxes of both stimuli. Indeed, SAFit2 treatment concentration dependently reduced the second capsaicin induced calcium influx, at 5 µM to less than half of the vehicle response (Fig. 7A, B). In contrast, the chiral analogue ddSAFit2 did not affect the capsaicin induced calcium influx (Fig. 7C, D).

To further investigate this effect of SAFit2, we performed additional control calcium imaging experiments with FKBP inhibitors from a different chemical series to verify whether this effect is FKBP dependent (Fig. 7E, F). However, none of the structurally unrelated FKBP inhibitors was capable of reducing the TRPV1 channel-mediated calcium fluxes (Fig. 7F). Since the structurally unrelated FKBP inhibitors were shown to potently occupy FKBP51 in human cells [16], we concluded that SAFit2 reduces the activity of TRPV1 in an FKBP51-independent manner.

As the TRPA1 channel is closely related to the TRPV1 channel and co-expressed in a subset of TRPV1 expressing neurons, we also examined the influence of SAFit2 on the TRPA1 channel. However, we did not observe any effect of SAFit2 on the activity of the TRPA1 channel (Additional file 1: Fig. S5).

To examine the impact of SAFit2 on the downstream signaling of TRPV1, we performed a calcitonin gene-related peptide (CGRP) assay. CGRP is a neuropeptide that is released from sensory neurons upon increasing intracellular calcium concentrations and is involved in pain mediation (Fig. 7G). Furthermore, the activation of its receptors can cause vasodilation and neurogenic inflammation [3, 15, 56]. Interestingly, we detected that SAFit2 reduces the CGRP concentrations in the supernatant of capsaicin-treated sensory neurons when they were co-treated with SAFit2 during stimulation (Fig. 7H). Based on these results, we conclude that the influence of SAFit2 on the TRPV1 channel activity also affects the downstream signaling events such as the secretion of CGRP.

Next, we wanted to determine whether SAFit2 antagonizes the TRPV1 directly. This might be problematic since TRPV1 antagonists led to severe side effects when they were tested in human volunteers [59]. Therefore, we assessed the influence of SAFit2 in a heterologous expression system using calcium imaging on TRPV1 transfected HEK-293t cells. There was no indication for TRPV1 inhibition over the large concentration range of 0.15 to 15 µM SAFit2 (Additional file 1: Fig. S6). In addition, we analyzed specifically whether SAFit2 has a direct impact on the channel activity of TRPV1 using whole-cell patch-clamp recordings. In these measurements, we demonstrated that 5 µM SAFit2 alone does not alter the amplitude of capsaicin-activated TRPV1 currents or the kinetics of the channel when added either to the intracellular or extracellular solution (Additional file 1: Fig. S7), ruling out a direct effect of SAFit2 on TRPV1 activity. In summary, SAFit2 does not inhibit the TRPV1 channel but desensitizes TRPV1 by affecting another factor that changes TRPV1 activity in sensory neurons.

**Fig. 8:**
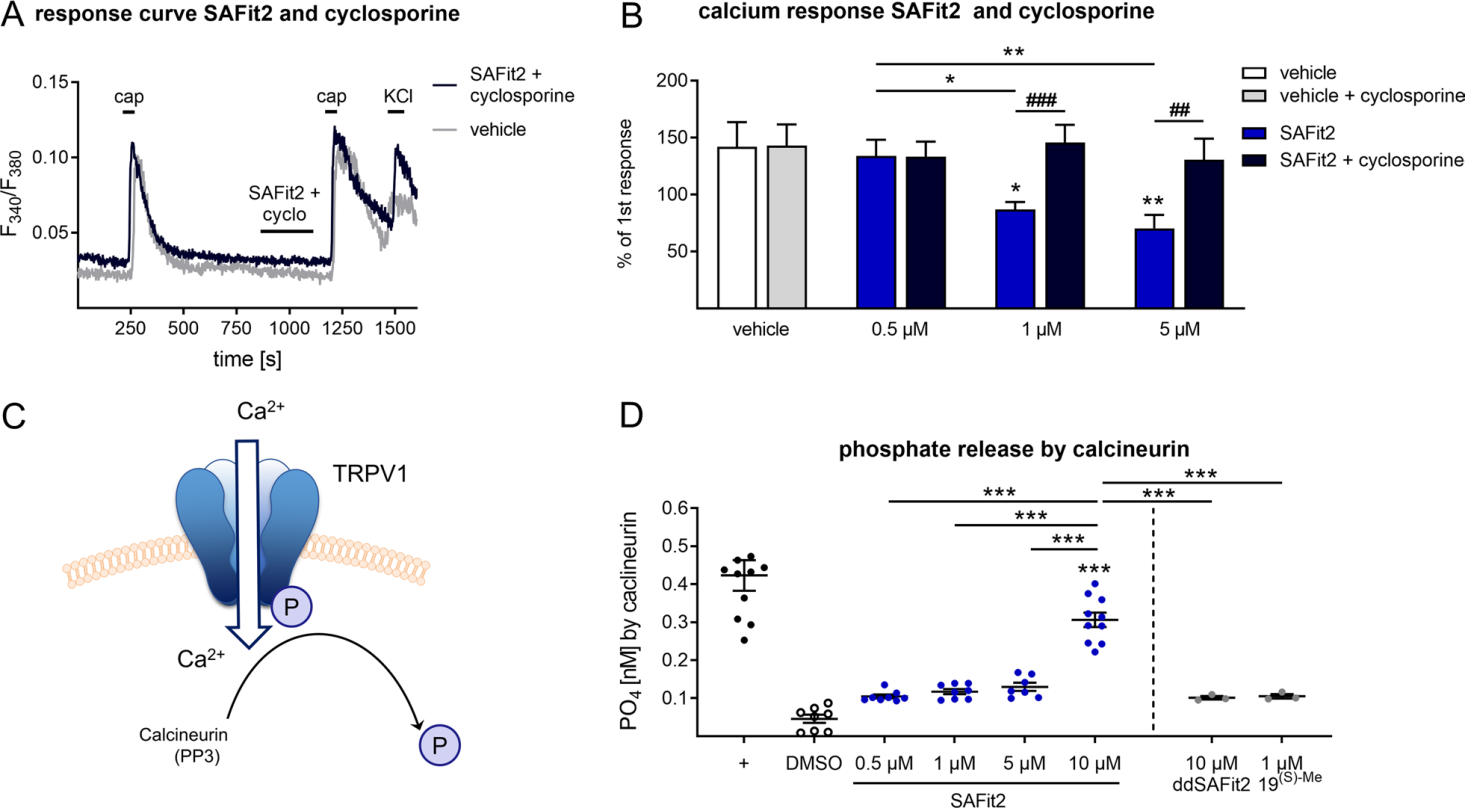
SAFit2 enhances the activity of the phosphatase calcineurin in primary sensory neurons. **A** Representative traces of calcium influxes in sensory neurons, which were pre-incubated with SAFit2 and cyclosporine (200 nM, 2 min) and stimulated with capsaicin (100 nM, 30 s) afterwards and KCl (50 mM) as a positive control for neuronal response at the end of each experiment. **B** Quantification of the calcium response with treatment related to the calcium response without treatment and comparison of SAFit2 treatment alone to SAFit2 and cyclosporine treatment. **C** Schematic illustration of the phosphatase calcineurin dephosphorylating the TRPV1 channel. The scheme was created with images from motifolio. **D** Quantification of the phosphate release after SAFit2 treatment in a calcineurin activation assay. **B** The data represent the mean ± SEM of 25–35 sensory neurons per group. *p < 0.05, **p < 0.01 one-way ANOVA with Tukey´s post hoc test, ^#^*p* < 0.05, ^##^*p* < 0.01 Student’s t-test with Welch´s correction. **D** The data represent the mean ± SEM of *n* = 3–11 per group. ***p < 0.001 one-way ANOVA with Sidak´s post hoc test. *TRPV1* transient receptor potential cation channel subfamily V member 1, *SAFit2* selective antagonist of FKBP51 by induced fit 2, *cap* capsaicin, *cyclo* cyclosporine, *KCl* potassium chloride

As a next step, we tried to examine how the desensitization of TRPV1 is mediated. Since the TRPV1 channel´s open probability is also strongly affected by phosphorylation [34], we suggested that SAFit2 probably has an impact on the most relevant phosphatase in sensory neurons which is the protein phosphatase 3 (PP3/calcineurin). Based on this, we hypothesized that SAFit2 enhances the calcineurin-mediated dephosphorylation of TRPV1 which further leads to a reduced open probability and a reduced calcium influx. To investigate the effect of SAFit2 on calcineurin, we performed calcium imaging with sensory neurons in which we inhibited calcineurin with cyclosporine while treating the cells with SAFit2 (Fig. 8A). Interestingly, we detected that an additional cyclosporine treatment restored calcium flux to vehicle level (Fig. 8B), indicating that SAFit2 may have an effect on the phosphatase activity of calcineurin. Finally, we explored this effect in a calcineurin activation assay in which we measured the phosphate release by calcineurin in the presence of different SAFit2 concentrations. Indeed, we observed an increased phosphate release by calcineurin with increasing SAFit2 concentrations (Fig. 8D). In contrast, the inactive analogue ddSAFit2 as well as structurally unrelated FKBP inhibitor did not enhance the phosphate release by calcineurin (Fig. 8D). Based on these results, we assumed that SAFit2 desensitizes the TRPV1 channel in a calcineurin-dependent manner, which is not mediated by FKBP51, and thereby is probably capable to counteract the increased activity of sensory neurons in neuroinflammation.

## Discussion

**Fig. 9:**
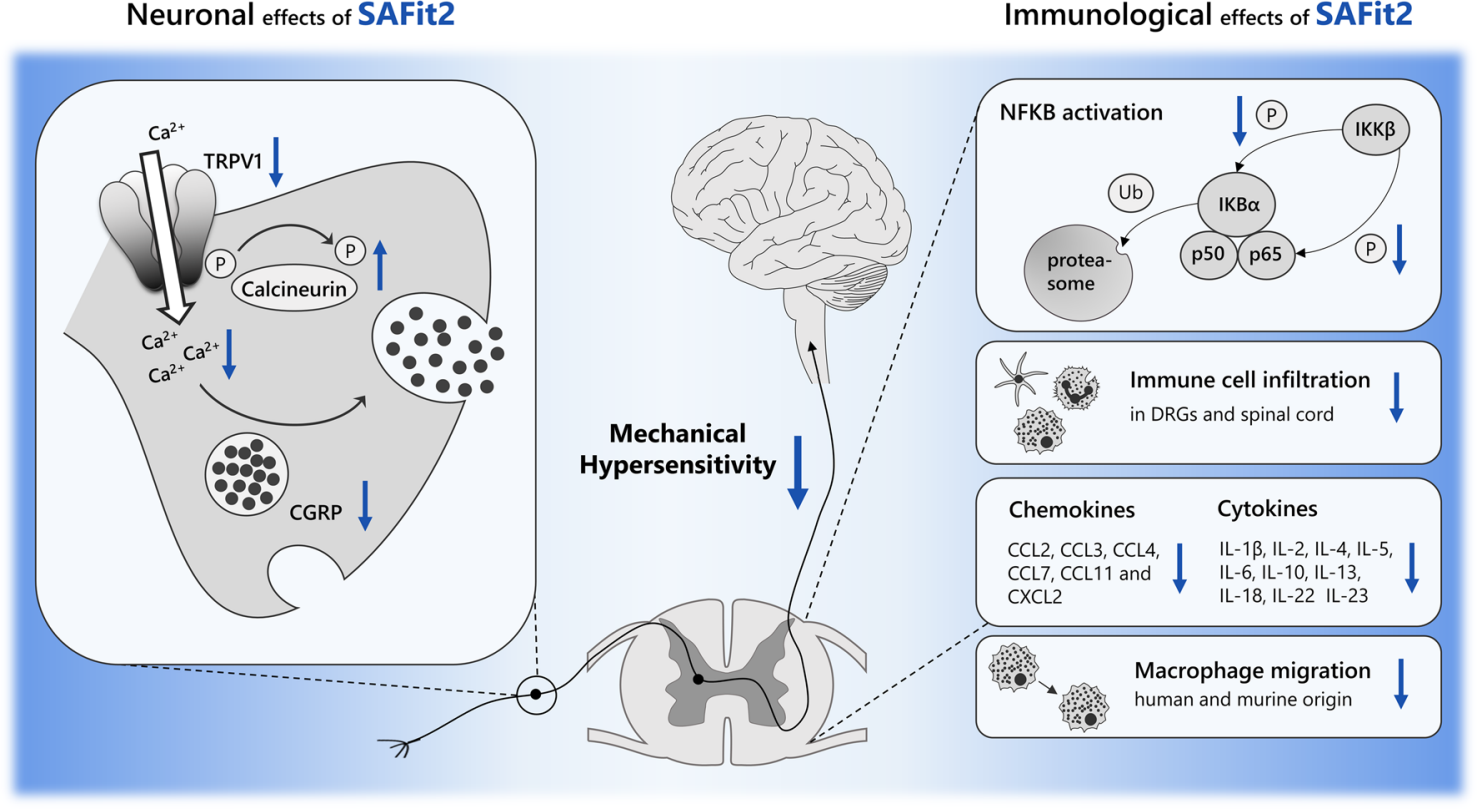
Summary scheme of the neuronal and immunological effects of SAFit2. SAFit2 reduced the mechanical hypersensitivity of nerve-injured mice by enhancing the activity of the phosphatase calcineurin which diminishes the open probability of the TRPV1 channel and subsequent the release of the neuropeptide CGRP. On the immunological side SAFit2 reduces the activation of the NF-κB signaling pathway since it reduces the phosphorylation of the inhibitor complex IκBα and the p65 itself. In addition, SAFit2 reduces the infiltration of immune cells into DRGs and spinal cord as well as chemokine and cytokine levels in the respective tissues. In vitro, we also could confirm that SAFit2 reduces the migration of human and murine macrophages. The scheme was created with images from motifolio. *SAFit2* selective antagonist of FKBP51 by induced fit 2, *TRPV1* transient receptor potential cation channel subfamily V member 1, *CGRP* Calcitonin gene-related peptide, *DRGs* dorsal root ganglia, *SC* spinal cord, *IκBα* NF-κB inhibitor alpha, *IKKβ* IκB kinase beta

SAFit2 has previously been shown to pass the blood–brain barrier [13]. Based on these results, it is quite likely that SAFit2 can also pass the blood spinal cord barrier and may partly mediate its analgesic effect in the central nervous system. As depicted in Fig. 9, we detected that SAFit2 reduces nerve injury-induced mechanical hypersensitivity in vivo by reducing neuroinflammation in neuronal tissue after SNI. On the one hand, SAFit2 has a direct effect on sensory neurons, particularly on the pain-mediating TRPV1 channel, on the other hand it reduces inflammatory processes. Thereby, SAFit2 diminishes enhanced neuronal activity and excessive neuroinflammation that can lead to central sensitization and to enhanced mechanical hypersensitivity. In detail, we showed that SAFit2 enhances the activity of the most relevant phosphatase in sensory neurons which is calcineurin. As a consequence, the phosphorylation state of the pain-mediating TRPV1 channel is decreased, and its open probability reduced. This in turn decreases the calcium influx and subsequently the transmitter release, including the release of the pro-inflammatory neuropeptide CGRP, leading to a reduction of exacerbated pain transmission (Fig. 9).

Moreover, we observed that SAFit2 ameliorates neuroinflammation in nerve-injured mice by reducing the infiltration of immune cells into neuronal tissue without affecting the inflammation resolution at the site of injury. This effect was further confirmed by the reduction of murine and human macrophage migration in vitro, pointing out translational potential to the human disease state. In addition, we detected that SAFit2 reduces the activation of the NF-κB signaling pathway. Both effects of SAFit2 reduced cytokine and chemokine levels in DRGs and spinal cord that contribute to neurogenic inflammation and pain transmission. In summary, SAFit2 reduces neuroinflammation and ameliorates nerve injury-induced mechanical hypersensitivity (Fig. 9).

Interestingly, we observed that SAFit2 showed its anti-hyperalgesic effects after the treatment phase. We therefore assume that SAFit2 interferes rather in the maintenance of neuropathic pain than in its onset, and that it takes a certain time until its cellular and molecular effects are reflected in behavioral changes.

This raises the question whether SAFit2 may serve as preventative or protective therapy for nerve injury-indued neuropathic pain. Mechanistically, we would argue that SAFit2 is a treatment option after injury rather than a protective/preventative therapeutic, because for the observed effects of SAFIt2 (reduction of nociceptive input and reduction of immune cell migration and mediator secretion) a previous injury is required for their initiation. It seems that SAFIt2 reduces these effects, but does not completely stop them. Therefore, we presume that SAFit2 can reduce these injury-related effects but might not be able to prevent them. However, this is speculation and should be addressed by future studies.

Neuroinflammation comprises the activation of resident immune cells, such as microglia and astrocytes, as well as migration of neutrophils, macrophages and T cells. Moreover, the secretion of pro-inflammatory mediators is a hallmark of neuroinflammation. These mediators can be cytokines, chemokines and growth factors and lead to central sensitization and an increased activity of peripheral sensory neurons, attributing these mechanisms an essential role in the onset and maintenance of neuropathic pain [21]. In this study, we detected that SAFit2 treatment can reduce the number of immune cells in the peripheral nervous system and at the interface between the peripheral and central nervous system. Likewise, the concentrations of pro-inflammatory and proalgesic mediators is reduced in the respective tissues compared to tissues from vehicle-treated mice. SAFit2 treatment also caused a reduction of anti-inflammatory cytokines (IL-4, IL-5 IL-13, IL-22) in DRGs and the spinal cord. These anti-inflammatory cytokines are mainly produced by T helper cells, such as Th2 and Th17 cells [10]. Although we did not directly measure the amount of Th cells by FACS, we see reduced amounts of other T cell species especially in the DRGs following SAFit2 treatment (Treg, Tm, Tγδ). Based on these data, we presume that T-cell migration to the DRGs is reduced after SAFit2 treatment, including Th2 and Th17 cells which leads to the observed decrease of IL-4, IL-5, IL13 and IL-22 concentrations. According to our observations, this is mediated by a reduced activation of the NF-κB signaling pathway in SAFit2-treated mice. The interaction between FKBP51 and the NF-κB pathway has previously been suggested in the context of steroid-refractory inflammation and cancer biology [24, 45] which is in line with our observations. In more detail, we mainly see strong influences of SAFit2 treatment on the phosphorylation state of p65 and IκBα, which are both central components of the NF-κB pathway.

We started the SAFit2 treatment 5 days after the SNI surgery, a time point when glial cell activation and neuroinflammation have already been initiated [53]. This might be the reason why the mechanical hypersensitivity of the SNI-treated animals is not completely restored to baseline levels and can explain why we still observe immune cells in DRGs and spinal cord, although significantly lower in the DRGs of SAFit2-treated animals. In addition, the difference of immune cell quantity is particularly strong between DRGs from vehicle and SAFit2-treated animals. We also observe a strong reduction of dendritic cells in DRG tissue after SAFit2 treatment. Recently, it was shown that dendritic cells release chemokines which cause an increased activity of peripheral sensory neurons via the activation of the C-C chemokine receptor 4 [48]. This is in line with our observation that SAFit2-treated animals show fewer dendritic cells and reduced levels of proalgesic mediators in DRGs.

In the spinal cord, the total number of immune cells is similar in SAFit2 and vehicle-treated mice, probably because the activated microglia and astrocytes in the spinal cord are resident immune cells, whereas infiltration of migrating immune cells occurs much stronger in the DRGs during persistent pain [42, 62]. However, we detected less eosinophils infiltrating into the spinal cord after SAFit2 treatment. Eosinophils are well known to induce several diseases like eosinophilia, eosinophilic vasculitis and many others when they are infiltrating into tissues [5, 35, 55]. Moreover, these cells were also associated with peripheral neuropathy and have been attributed effects on sensory nerve branching [36].

Apart from the immune cell component, neuroinflammation can also be initiated by increased neuronal activity and the subsequent release of neuropeptides, such as calcitonin gene-related peptide (CGRP) which serves as chemoattractant for immune cells [6, 7]. While most anti-inflammatory agents can target the immune cell component of neuroinflammation, they are generally not capable of addressing the neuronal component. However, we observed that SAFit2 causes a desensitization of the neuronal TRPV1 channel that seems to be mediated by a calcineurin-dependent dephosphorylation.

While TRPV1 represents an interesting target in persistent pain, its complex regulatory functions and its requirement for normal maintenance of the body temperature has made it difficult to target and even more than 20 years after its initial discovery, there is still no TRPV1 antagonist available for clinical use [23, 25, 59]. It may thus be more promising to keep TRPV1 activity in the physiological range and reduce its sensitization that occurs in pathophysiological pain states. In this regard, the desensitizing effect of SAFit2 could be an additional beneficial effect of the compound to ameliorate persistent pain. Subsequently, the release of the neuropeptide CGRP triggered by an enhanced TRPV1 channel activation can also be diminished by SAFit2 in cultured sensory neurons, which shows that SAFit2 can reduce neuroinflammation both at the immune cell level, but also at the level of sensory neurons and neuropeptide release.

A limitation of this study is the lack of female mice in the behavioral assays. Previously, it was reported that sex differences occur in development and maintenance of neuropathic pain in vivo [33, 51]. Although the contribution of FKBP51 to neuropathic pain has been found to be sex independent [30], it is conceivable that the effects of SAFit2 during neuropathic pain and in neuroinflammation differ between male and female mice.

Besides the influences of the drug target FKBP51 in the peripheral nervous system, it was previously also revealed as a target for central nervous system (CNS) indications and psychological disorders [46]. In particular, a strong genetic association was validated for FKBP51 in stress-related and endocrinologic mediated diseases as depression, type two diabetes and obesity [46]. Moreover, inhibiting FKBP51 recently revealed an improvement of anxiety related [18] as well as of stress-related disorders [40].

In the context of chronic pain, the target FKBP51 was already shown to be upregulated in spinal cord and DRGs, whereas the knockout of FKBP51 was shown to relieve pain in a chronic ankle joint inflammation model [31]. Moreover, the knockdown of FKBP51 was also shown to ameliorate neuropathic pain after chronic constriction injury [61], revealing FKBP51 as a promising therapeutically target for a broad range of disorders ranging from psychological disorders to chronic pain. However, the development of a possible drug candidate was hampered in the past, due to the lack of a specific and potent FKBP51 inhibitor, highlighting SAFit2 as a novel and promising treatment option for neuropathic pain.

## Supplementary Information

**Additional file 1: Figure S1.** Gene expression of neuronal stress and oxidative stress markers in lumbar DRGs and spinal cord of SAFit2 treated mice 21 days after SNI. **Figure S2.** Cytokine and chemokine levels in lumbar DRGs and spinal cord of SAFit2 treated SNI mice after 21 days. **Figure S3.** Synthesis of ddSAFit 12 and competitive fluorescence assay (FPA) of SAFit2 and ddSAFit2**. Figure S4.** Cytotoxic and metabolic influence of SAFit2 on primary bone marrow derived macrophages. **Figure S5.** SAFit2 has no impact on the TRPA1 activity in primary sensory neurons. **Figure S6.** SAFit2 has no direct impact on the human hTRPV1 channel in HEK-293t cells. **Figure S7.** Extracellularly or intracellularly administered SAFit2 has no effect on the amplitude and kinetics of capsaicin-activated TRPV1 currents in HEK-293 cells. **Figure S8.** Uncropped Western Blot images for NF-κB signaling pathway.

## Data Availability

All data generated or analyzed during this study are included in this published article and its supplementary information files.

## Abbreviations

BMDM: Bone marrow-derived macrophages
CCL: C-C motif ligand
CGRP: Calcitonin gene-related peptide
CXCL: C-X-C motif ligand
DRGs: Dorsal root ganglia
FKBP51: FK506-binding protein 51
IκBα: NF-κB inhibitor alpha
IKKβ: IΚBα kinase beta
IL: Interleukin
SAFit2: Selective antagonist of FKBP51 by induced fit
SC: Spinal cord
SNI: Spared nerve injury
TRP: Transient receptor potential
TRPV1: Transient receptor potential cation channel subfamily V member 1
