# Supplementary material for: SAFit2 reduces neuroinflammation and ameliorates nerve injury-induced neuropathic pain
Source: J Neuroinflammation. 2022 Oct 10;19:254. doi: 10.1186/s12974-022-02615-7 (PMC9552419; doi:10.1186/s12974-022-02615-7)
Supplement: Supplementary file 1 — Additional file 1: Figure S1. Gene expression of neuronal stress and oxidative stress markers in lumbar DRGs and spinal cord of SAFit2 treated mice 21 days after SNI. Figure S2. Cytokine and chemokine levels in lumbar DRGs and spinal cord of SAFit2 treated SNI mice after 21 days. Figure S3. Synthesis of ddSAFit 12 and competitive fluorescence assay (FPA) of SAFit2 and ddSAFit2. Figure S4. Cytotoxic and metabolic influence of SAFit2 on primary bone marrow derived macrophages. Figure S5. SAFit2 has no impact on the TRPA1 activity in primary sensory neurons. Figure S6. SAFit2 has no direct impact on the human hTRPV1 channel in HEK-293t cells. Figure S7. Extracellularly or intracellularly administered SAFit2 has no effect on the amplitude and kinetics of capsaicin-activated TRPV1 currents in HEK-293 cells. Figure S8. Uncropped Western Blot images for NF-κB signaling pathway. [file 12974_2022_2615_MOESM1_ESM.docx]

**Additional file 1**

**SAFit2 reduces neuroinflammation and ameliorates nerve injury-induced neuropathic pain**

Saskia Wedel^1^, Praveen Mathdoor^2^, Oliver Rauh^3^, Tim Heymann^4^, Cosmin I. Ciotu^5^, Dominik C. Fuhrmann^2^, Michael J. M. Fischer^5^, Andreas Weigert^2^, Natasja de Bruin^5^, Felix Hausch^4^, Gerd Geisslinger^1,6^ and Marco Sisignano^1,6*^

^1^ Institute of Clinical Pharmacology, *pharmazentrum frankfurt*/ZAFES, University Hospital, Goethe-University, 60590 Frankfurt am Main, Germany

^2^Institute of Biochemistry I, Faculty of Medicine, Goethe-University Frankfurt, 60590 Frankfurt am Main, Germany

^3^Membrane Biophysics, Department of Biology, Technical University of Darmstadt, 64287 Darmstadt, Germany

^4^Department of Chemistry, Technical University of Darmstadt, 64287 Darmstadt, Germany

^5^Center of Physiology and Pharmacology, Medical University of Vienna, 1090 Vienna, Austria

^6^Fraunhofer Institute for Translational Medicine and Pharmacology ITMP and Fraunhofer Cluster of Excellence for Immune Mediated Diseases CIMD, 60596 Frankfurt am Main, Germany

Figures: 8


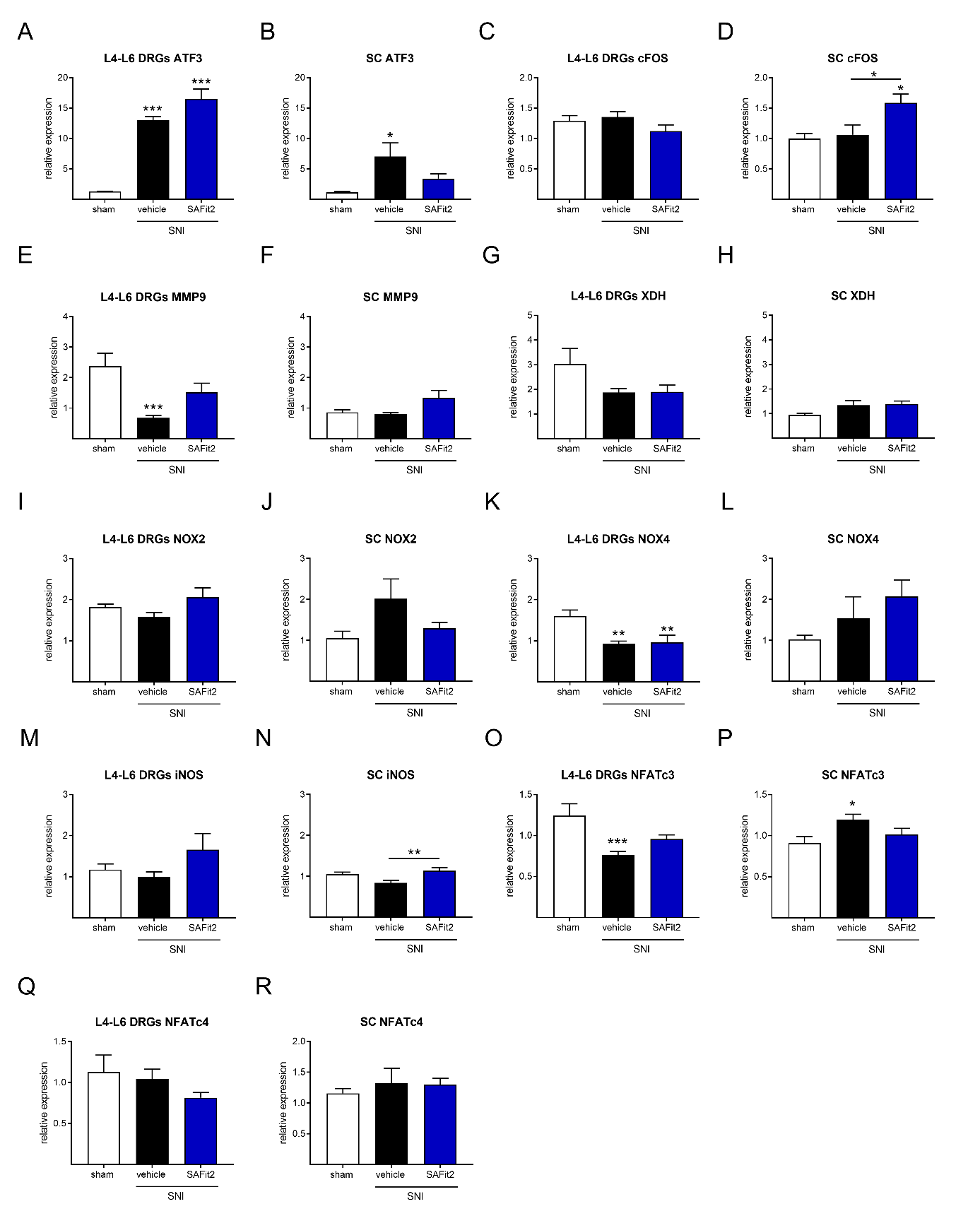


Figure S1: Gene expression of neuronal stress and oxidative stress markers in lumbar DRGs and spinal cord of SAFit2 treated mice 21 days after SNI. Mice underwent a SNI surgery and were then treated with either vehicle or 10 mg/kg SAFit2 from day five to ten after the surgery. After 21 days, the expression of neuronal stress markers: ATF3 (A,B), cFOS (C,D), MMP9 (E,F), oxidative stress markers: XDH (G,H), NOX2 (I,J), NOX4 (K,L), iNOS (M,N) and NFAT subunits: NFATc3 and NFATc4 (O-R) was measured. The data represents the mean ± SEM of 3-4 mice per group, measured in technical triplicates respectively. * p < 0.05, ** p < 0.01, *** p < 0.001 one-way ANOVA with Tukey´s post hoc test. Abbreviations: SAFit2: selective antagonist of FKBP51 by induced fit 2, SNI: spared nerve injury, ATF3: activating transcription factor 3, MMP9: matrix metallopeptidase 9, XDH: xanthine dehydrogenase, NOX2/4: NADPH oxidase 2/4, iNOS: inducible nitric oxide synthase, NFAT: nuclear factor of activated T cells


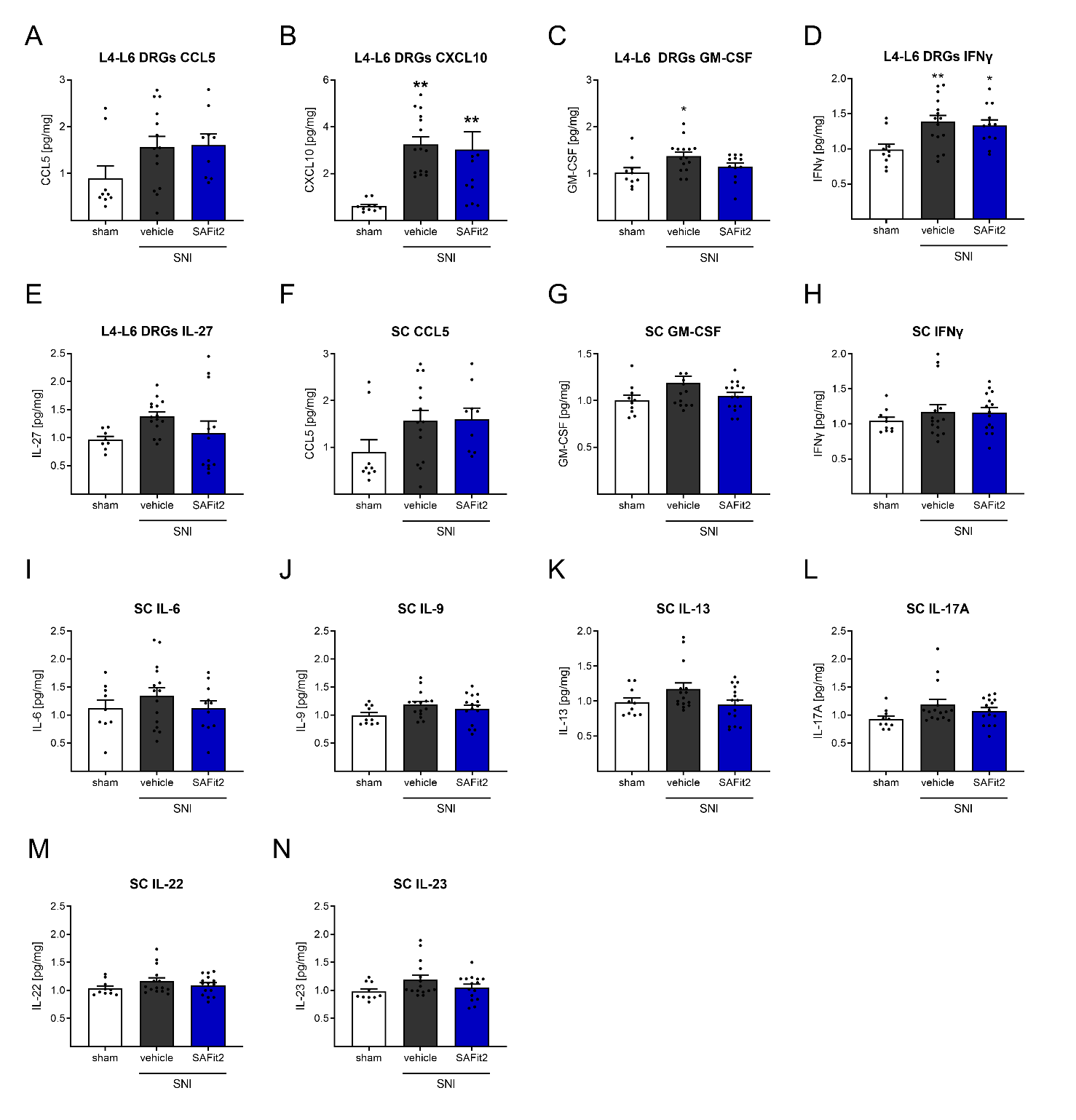


Figure S2: Cytokine and chemokine levels in lumbar DRGs and spinal cord of SAFit2 treated SNI mice after 21 days. Mice underwent SNI surgery and were then treated with either vehicle or 10 mg/kg SAFit2 from day five to ten after the surgery. After 21 days, L4-L6 DRGs and spinal cord were isolated from ipsilateral and contralateral sides with which lysates a multiplex immunoassay was performed including a panel of 26 cytokines and chemokines. Shown is a section of mediators measured in L4-L6 DRGs (A-E) and spinal cord (F-N) after SAFit2 treatment. The data represents the mean ± SEM from 5 mice per group, measured in technical triplicates respectively. The raw data was related to the total protein amount of the sample and the ipsilateral (injured) value was normalized to the contralateral (control) value per animal. * p < 0.05, ** p < 0.01, *** p < 0.001 one-way ANOVA with Tukey´s post hoc test. Abbreviations: SNI: spared nerve injury, DRGs: dorsal root ganglia, SC: spinal cord, SAFit2: selective antagonist of FKBP51 by induced fit 2


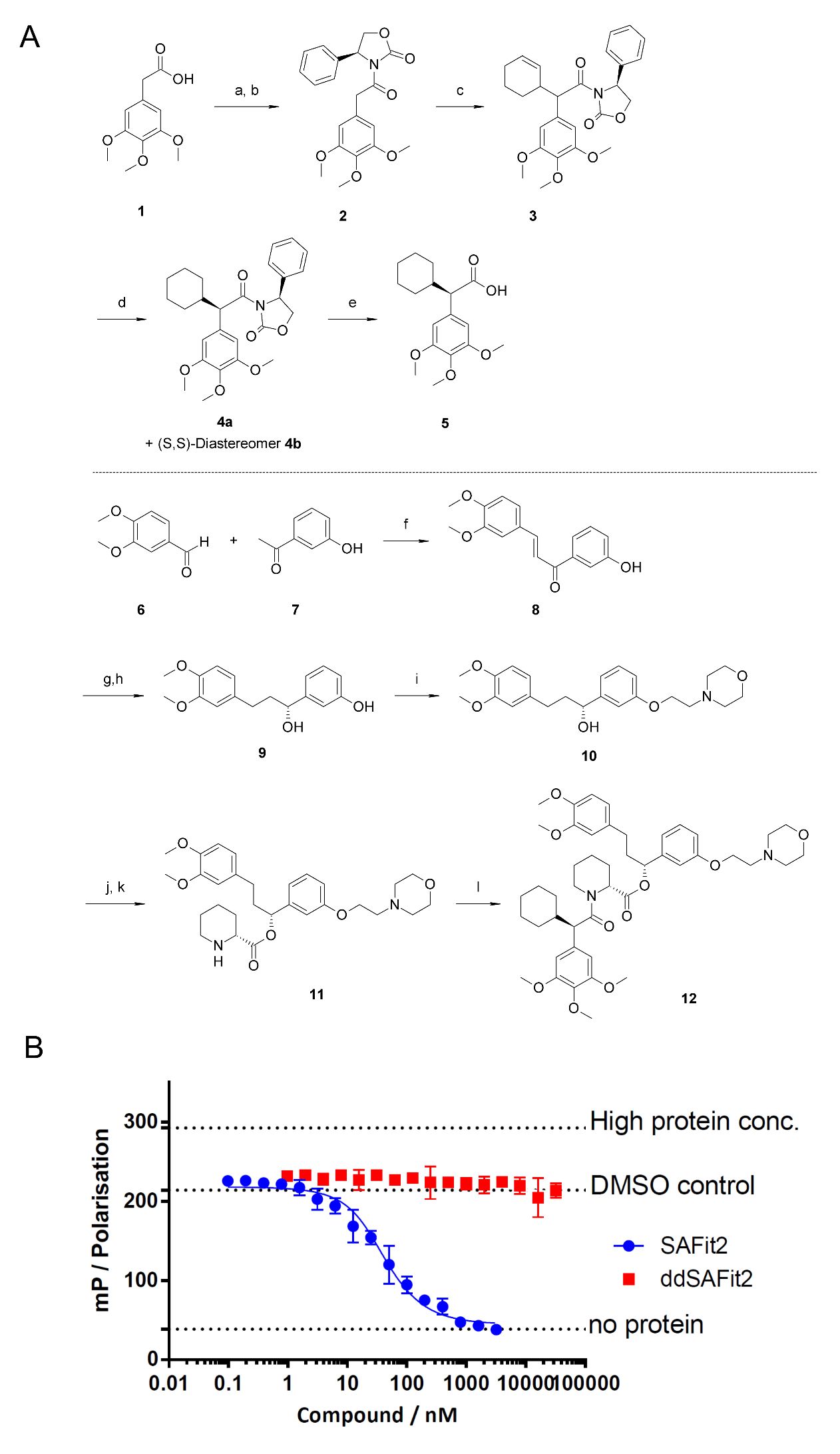


**Figure S3: Synthesis of ddSAFit 12 and competitive fluorescence assay (FPA) of SAFit2 and ddSAFit2. (A)**. **a)** Pentafluorphenol, EDC・HCl, DMAP, DCM 0 °C, 74% yield, **b)** (S)-4-phenyloxazolidin-2-one, n-BuLi, THF,−78 °C, 76% yield, **c)** LiHMDS, 3-bromo cyclohexene, THF -78 °C, 74% yield, **d)** 1 bar H_2_, Pd/C, MeOH, 19% of 4a and 70% of the diastereomer 4b, **e)** LiOH/H_2_O_2_, THF/H_2_O 8:5, 0 °C, 69% yield, **f)** KOH, H_2_O/EtOH, 0 °C, 99% yield, g) Zn/NH_4_Ac, MeOH, r.t., 84% yield, **h)** atmospheric H_2_, KOtBu, Noyori catalyst, IPA, r.t., 95%, **i)** K_2_CO_3_, 2-chloroethylmorpholine hydrochloride, MeCN, reflux, 78% yield, **j)** EDC・HCl, DMAP, R)-1-(tert-butoxycarbonyl)piperidine-2-carboxylic acid, DCM, 0 °C, 78% yield, **k)** DCM/TFA 2:1, r.t., quant. yield, **l)** HATU, 4a, DiPEA, DCM/DMF, r.t., 78% yield. **(B)** Competitive fluorescence polarization assay (FPA) of SAFit2 (blue dots) and ddSAFit2 (red squares), high protein control, DMSO control, and no protein control are indicated by a tick mark and dashed lines. Fitting the curve results in a K_d_ of 8 nM for SAFit2 and > 80000 nM for ddSAFit2. FPA was performed as described previously (Bauder et al., 2021 (https://doi.org/10.1021/acs.jmedchem.0c02195).


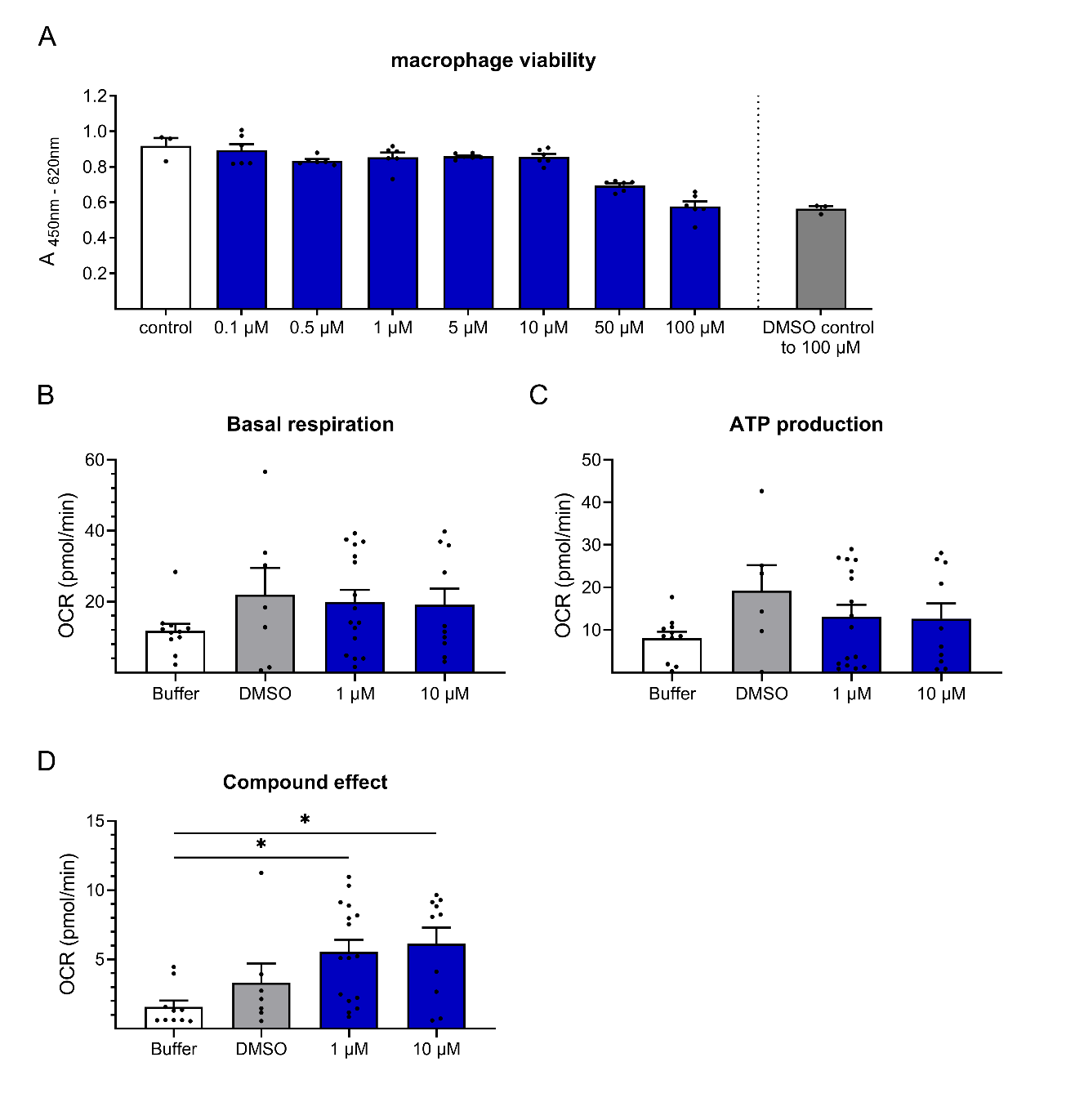


Figure S4: Cytotoxic and metabolic influence of SAFit2 on primary bone marrow derived macrophages. (A) The effect of SAFit2 on the macrophage viability was assessed in a WST-1 assay showing that SAFit2 has no influence on the cell viability. The data represents the mean ± SEM of 3-6 per group (B-D) The influence of SAFit2 the macrophage metabolism was analyzed with a Seahorse bioanalyzer measuring the oxygen consumption rate (OCR). SAFit2 affects neither the basal respiration (B) nor the ATP production (C) of macrophages. It slightly increases the total respiration of macrophages compared to the buffer control, which is negligible since the DMSO control is also slightly increased. (D). The data represents the mean ± SEM of 1-2 runs per group, measured in several technical replicates respectively. * p < 0.05 one-way ANOVA with Tukey´s post hoc test.


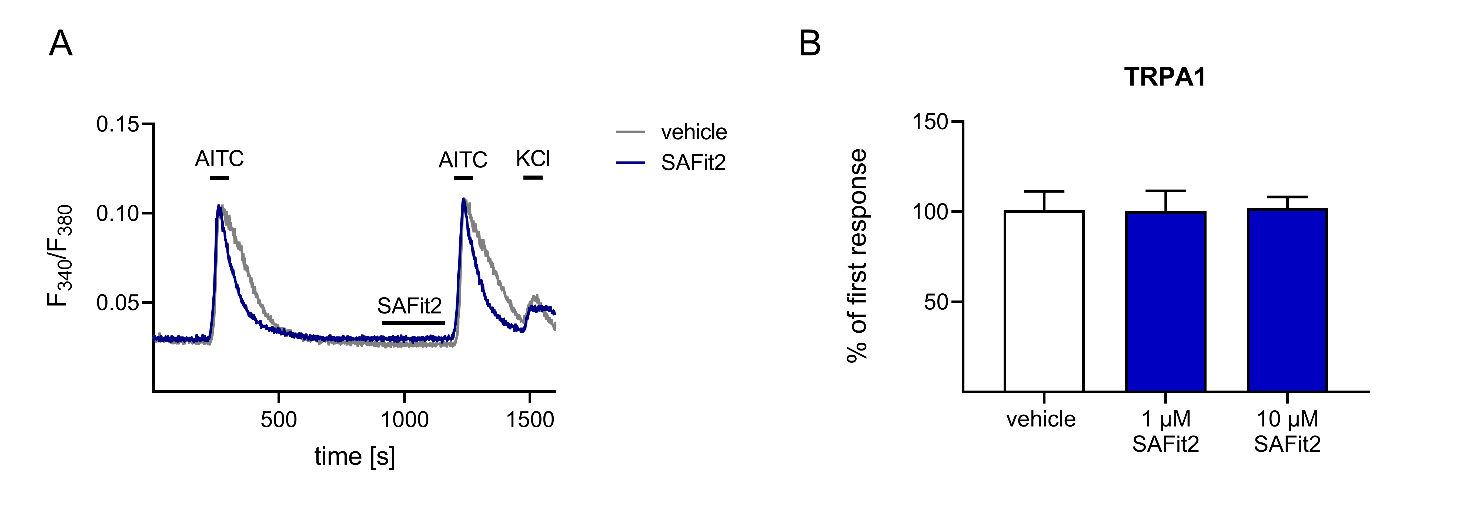


Figure S5: SAFit2 has no impact on the TRPA1 activity in primary sensory neurons. Primary sensory neurons were isolated from mice and the effect of SAFit2 on the TRPA1 channel activity was assessed in calcium imaging experiments. (A) Representative traces of TRPA1 calcium fluxes from sensory neurons, which were pre-incubated with SAFit2 for 2 minutes and stimulated with the gold standard TRPA1 agonist allyl isothiocyanate (AITC) (100 µM, 45 s) afterwards and KCl (50 mM) as a positive control for neuronal response at the end of each experiment. (B) Quantification of the treated calcium response (second stimulus) related to the untreated calcium response (first stimulus). The data represents the mean ± SEM of 37-51 sensory neurons per group.


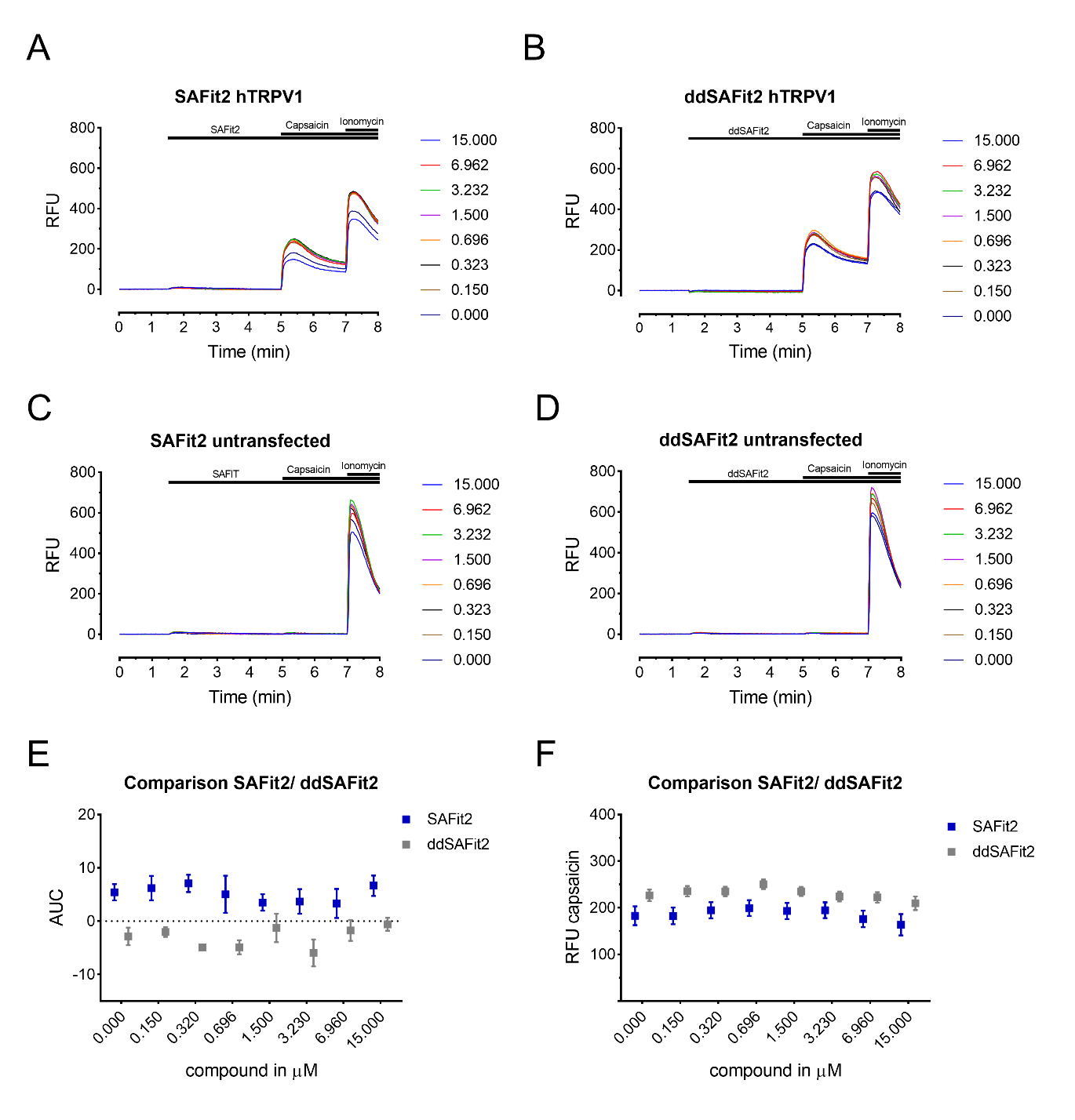


Figure S6: SAFit2 has no direct impact on the human hTRPV1 channel in HEK-293t cells. (A, B) Mean relative fluorescence intensity (RFU) traces of hTRPV1 transfected HEK-293t cells after the treatment with up to 15 µM SAFit2 (A) and ddSAFit2 (B), followed by a stimulation with 200 nM capsaicin and 2 µM ionomycin as a positive control. (C, D) Mean relative fluorescence intensity (RFU) traces of untransfected HEK-293t cells after the treatment with up to 15 µM SAFit2 (C) and ddSAFit2 (D), followed by a stimulation with 200 nM capsaicin and a positive control with 2 µM ionomycin. (E) Shown is the comparison of the total area under the curve (AUC) values of SAFit2 and ddSAFit2 for respective concentrations. (F) Shown is the calculated RFU with its error for capsaicin stimulations after SAFit2 and ddSAFit2 treatment with respective concentrations. The data represents the mean ± SD of 3 measurements.


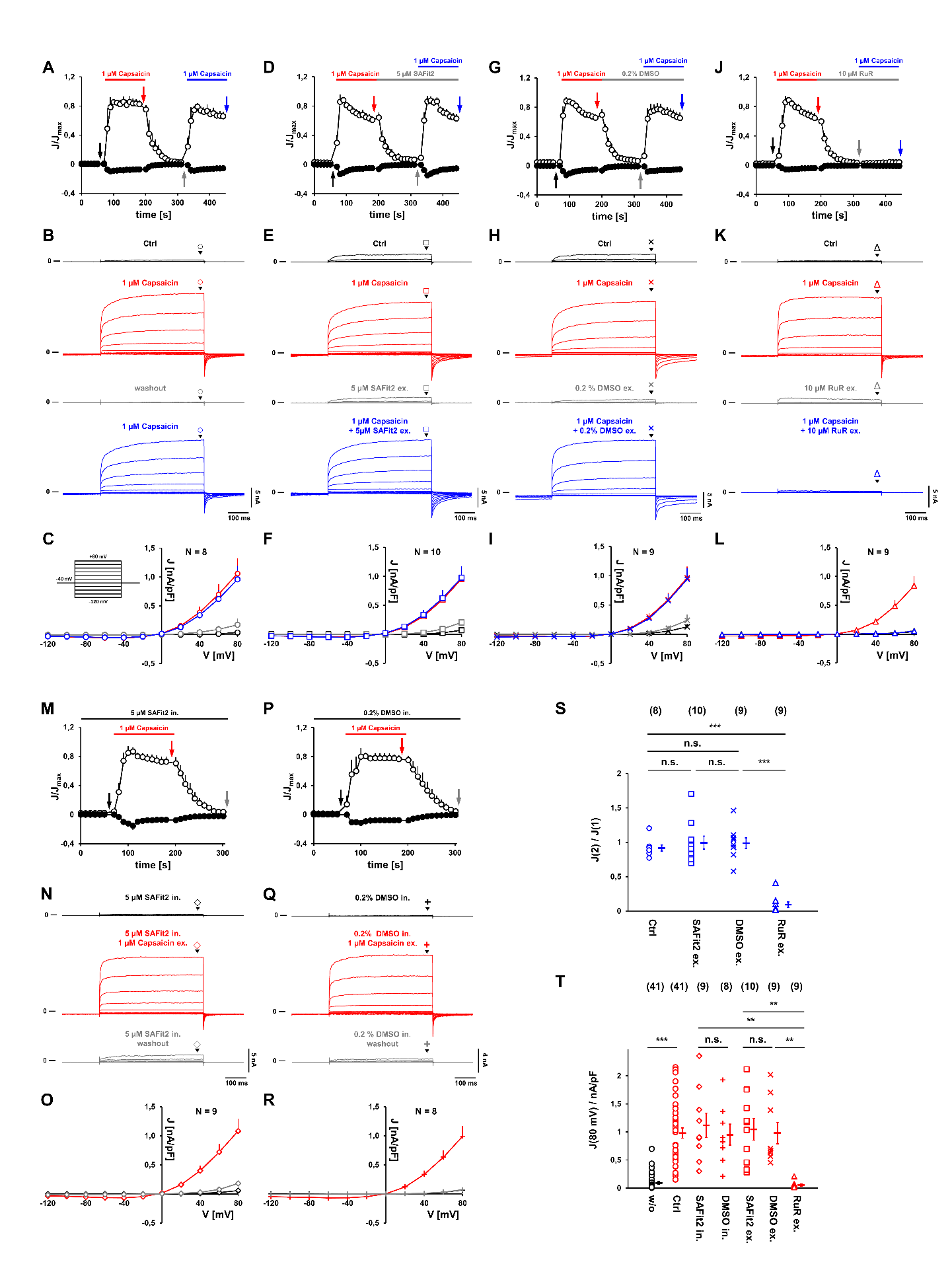


Figure S7: Extracellularly or intracellularly administered SAFit2 has no effect on the amplitude and kinetics of capsaicin-activated TRPV1 currents in HEK-293 cells. Whole-cell patch-clamp experiments were performed on HEK-293 cells transiently expressing TRPV1-GFP. TRPV1 currents were activated by addition of 1 µM capsaicin to the bath solution. (A-L) To determine the effect of extracellularly added compounds on TRPV1 activation, capsaicin was applied twice for two minutes each: the first application was used to determine TRPV1 activity in the absence, the second in the presence of the compound. The compound was administered during washout after the first capsaicin application by perfusion with bath solution. The following compounds were tested: no compound (A-C), SAFit2 (D-F), DMSO (vehicle control) (G-I), and Ruthenium Red (RuR, positive control) (J-K). (A,D,G,J) Time courses of normalized current densities at -60 mV (closed circles) and +60 mV (open circles) as obtained by repeatedly applying 500 ms voltage ramps from -60 mV to +60 mV. Current densities were normalized for each cell to the maximal current density measured at +60 mV during the first capsaicin application. Data points represent arithmetic mean ± SEM of N = 4-10 cells. (B,E,H,K) Representative whole-cell current traces evoked by the voltage-step protocol shown in (C). The time points at which the voltage-step protocol was applied are indicated in the time courses in A,D,G,J with arrows of the corresponding color. (C,F,I,L) Steady-state current densities obtained from voltage-clamp measurements as shown in B,E,H,K. The time points at which the current densities were measured are highlighted in B,E,H,K with the corresponding symbols. Data points represent arithmetic mean ± SEM. The number of measured cells N is indicated in the graphs. (M-R) To determine whether SAFit2 can directly affect TRPV1 activity from the intracellular side of the plasma membrane, patch-clamp experiments were performed with 5 µM SAFit2 (M-O) and with 0.2% DMSO (vehicle) added to the pipette solution (P-R). (M,P) Time courses of normalized current densities at -60 mV (closed circles) and +60 mV (open circles) as obtained by repeatedly applying 500 ms voltage ramps from -60 mV to +60 mV. Current densities were normalized for each cell to the maximal current density measured at +60 mV. Data points represent arithmetic mean ± SEM of N = 5-9 cells. (N,Q) Representative whole-cell current traces evoked by the voltage-step protocol shown in (C). The time points at which the voltage-step protocol was applied are indicated in the time courses in (M,P) with arrows of the corresponding color. (O,R) Steady-state current densities obtained from voltage-clamp measurements as shown in (N,Q). The time points at which the current densities were measured are highlighted in (N,Q) with the corresponding symbols. Data points represent arithmetic mean ± SEM. The number of measured cells in N is indicated in the graphs. (S) Ratios J(2)/J(1) of steady-state current densities measured at +80 mV at the end of the first (J(1)) and second (J(2)) capsaicin application in the voltage-clamp experiments shown in (A-L). (T) Steady-state current densities at +80 mV obtained from the voltage-clamp experiments shown in (A-R) without (w/o, black circles) and after 2 minutes of capsaicin application (red symbols) to the bath solution in the absence (Ctrl) or presence of 5 µM SAFit2, 0.2% DMSO (vehicle) or 10 µM ruthenium red (RuR) in the pipette (in.) or bath solution (ex.). (S) and (T) show the values of the individual measurements as well as the arithmetic mean ± SEM. The number of measured cells is given in brackets above each group. Not significant (n.s.) p ≥ 0.05, * p < 0.05, ** p < 0.01, *** p < 0.001 student t-test with Welch´s correction.


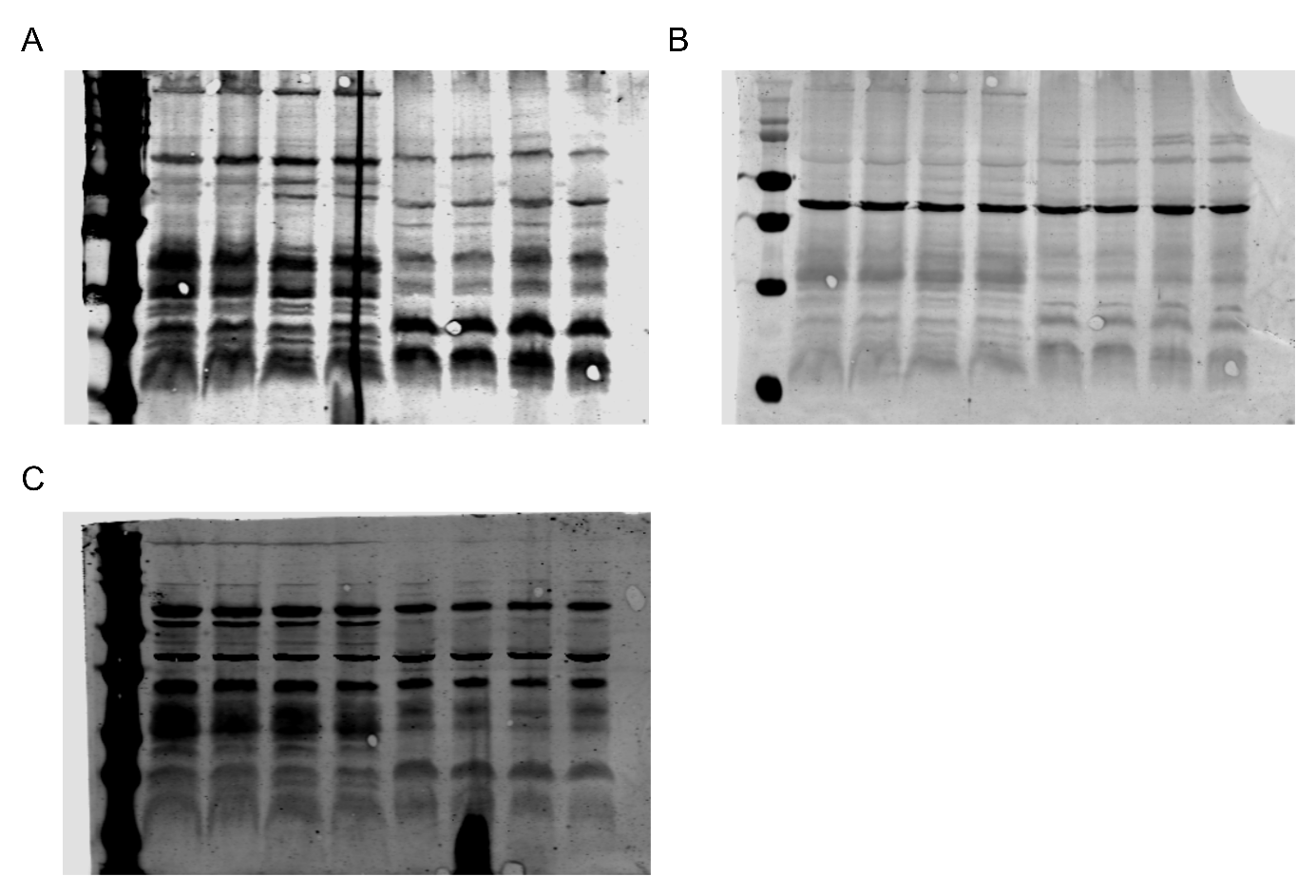


**Figure S8: Uncropped Western Blot images for NF-κB signaling pathway.** Images of **(A)** phosphorylated p65 and IκBα, **(B)** β-Actin loading control for phosphorylated proteins and **(C)** total protein amount of IKKβ, p65, IκBα and the respective loading control β-Actin.

**Methods for the synthesis of ddSAFit2**

If not mentioned otherwise, reactions were performed in an oven dried flask under argon atmosphere. ^1^H- and ^13^C-NMR spectra were recorded at the NMR department of chemistry of the Technical University of Darmstadt (TUD) on a Bruker DRX500 and at the Thiele lab by Johann Primozic and Matthias Brauser on a Bruker Avance III 600 MHz and Bruker Avance III HD 700 MHz spectrometer. Chemical shifts for ^1^H and ^13^C are given in ppm (δ) relative to the tetramethylsilane (TMS) internal standard. Deuterated chloroform (CDCl_3_ was used as solvent and the spectra calibrated according to their corresponding peak. The multiplicities are abbreviated as follows: singlet (s), doublet (d), triplet (t), quartet (q), doublet of doublets (dd), multiplet (m). HRMS spectra were acquired on an LTQ Orbitrap XL that was calibrated using the calmix solution of the manufacturer at least a week before the measurements were conducted. Chiral HPLC was performed using a Beckman System Gold 125S Solvent Module with a Beckman System Gold Diode Array Detector Module 168 recording UV spectra at 220 nm using a Daicel chemical industries Ltd, Chiralcel OD-H, normal phase analytical column, 250 × 4.6 mm, 5 µm with an isocratic gradient given for each compound using n heptane as solvent A and isopropyl alcohol (IPA) as solvent B. UHPLC-MS measurements were performed on an Agilent 1260 infinity II system consisting out of a flexible pump, a Vialsampler, a multicolumn compartment with a column oven, a DAD detector, and a 6125B MSD. Data was acquired with a 50 x 2.1 mm, 1.9 µm EC-C18 Poroshell 120 column using a gradient starting with an isocratic hold of 0.2 min at 5% solvent B up to 100% solvent B over 1.8 min followed by a hold for 1 min with solvent A being water with 0.1% formic acid and solvent B acetonitrile with 0.1% formic acid. Purity is determined by UV detection at 220 nm and given in percent. LC-MS measurements were performed using a Beckman System Gold 125 Solvent Module and Beckman System Gold 199 Detector Module with a YMC Pack Pro C8, 100 × 4.6 mm, 3 µm column using a 0 - 100% B gradient in 19 min with a 1 mL/min flow using solvent A: 95% H_2_O, 5% MeCN, 0.1% formic acid and solvent B: 95% MeCN, 5% H_2_O, 0.1% formic acid with a LCQ Deca XP Plus operated in ESI mode. Flash chromatography is performed using an Isolera system from Biotage using the flash columns of the manufacturer with DAD-UV detection. If not mentioned otherwise, separations are performed with a linear 10 column volume gradient. Manual column chromatography was performed using Kieselgel 60 from Roth, 0.04 − 0.063 mm with the solvent mixture mentioned for each compound. Preparative HPLC is performed on an Interchim Puriflash 5.250 system with UV detection. Purifications are performed using a Luna 250 x 21.2 mm, 5 µm C18 column with a 100 Å pore size using a gradient from 5% B to 100% B in ten column volumes at a flow rate of 30 mL/min unless mentioned otherwise. Solvent A is water with 0.1 TFA and solvent B acetonitrile with 0.1% TFA.

**(E)-3-(3,4-Dimethoxyphenyl)-1-(3-hydroxyphenyl)prop-2-en-1-one (8):** 9.0 g (54.2 mmol, 1.0 eq) 3,4-Dimethoxybenzaldehyde and 7.37 g (54.2 mmol, 1.0 eq) 3-Hydroxyacetophenone are dissolved in 90 mL EtOH and the mixture is cooled to 0 °C. 24.5 g (217 mmol, 4.0 eq) potassium hydroxide are dissolved in 60 mL water and to the aforementioned mixture. The mixture is stirred for 2 h and then quenched by adding ice and then 3 M HCl until a pH of 2 is reached. The mixture is crystallized from the quenched reaction mixture to yield 15.3 g (99%) as a yellow solid. ^1^H-NMR (500 MHz, CDCl_3_): δ 7.77 (dd, J = 15.6, 1.4 Hz, 1H), 7.67 – 7.59 (m, 1H), 7.56 (d, J = 7.7 Hz, 1H), 7.40 – 7.33 (m, 2H), 7.22 (dt, J = 8.4, 1.8 Hz, 1H), 7.15 (t, J = 1.8 Hz, 1H), 7.13 – 7.07 (m, 1H), 6.89 (dd, J = 8.3, 1.5 Hz, 1H), 6.64 (s, 1H), 3.94 (s, 3H), 3.93 (s, 3H). ^13^C-NMR (126 MHz, CDCl3): δ 190.9, 156.6, 151.7, 149.4, 145.7, 139.9, 130.0, 127.9, 123.5, 120.9, 120.3, 120.0, 115.3, 111.3, 110.3, 56.2, 56.1. UHPLC-MS: t_R_ = 1.753 min (99%), m/z: calculated = 285.11 [M+H]^+^, found = 285.2 [M+H]^+^.

**3-(3,4-Dimethoxyphenyl)-1-(3-hydroxyphenyl)propan-1-one:** 14.0 g (215 mmol, 10 eq) zinc and 16.5 g (215 mmol, 10 eq) ammonium acetate are suspended in 100 mL MeOH. 6.1 g (21.5 mmol, 1.0 eq) **8** are dissolved in 100 mL MeOH and added dropwise to the aforementioned mixture. The solids are filtered, washed with MeOH and the crude product is precipitated from the filtrate by addition of water. The crude product is purified by recrystallization from water/methanol to yield 5.17 g (84%) as a white solid. ^1^H-NMR (500 MHz, CDCl_3_): δ 7.54 (dd, J = 2.7, 1.6 Hz, 1H), 7.49 (dq, J = 7.9, 1.2 Hz, 1H), 7.31 (td, J = 8.0, 1.1 Hz, 1H), 7.08 (ddd, J = 8.0, 2.6, 0.9 Hz, 1H), 6.83 – 6.73 (m, 3H), 6.63 (d, J = 42.6 Hz, 1H), 3.84 (s, 3H), 3.84 (s, 3H), 3.26 (dd, J = 8.3, 6.9 Hz, 2H), 3.05 – 2.97 (m, 2H). ^13^C-NMR (126 MHz, CDCl_3_): δ 200.4, 156.6, 149.0, 147.5, 138.3, 133.9, 130.0, 120.8, 120.6, 120.3, 114.7, 112.1, 111.6, 56.1, 56.0, 40.9, 30.0. UHPLC-MS: t_R_ = 1.778 min (98 %), m/z: calculated = 287.13 [M+H]^+^, found = 287.2 [M+H]^+^.

**(R)-3-(3-(3,4-Dimethoxyphenyl)-1-hydroxypropyl)phenol (9):** 9.70 g (33.9 mmol, 1 eq) **3-(3,4-Dimethoxyphenyl)-1-(3-hydroxyphenyl)propan-1-one** and 50.8 mL (50.8 mmol, 1.5 eq) 1 M potassium butoxide were dissolved in 100 mL IPA. The mixture was degassed with Ar and 270 mg (0.27 mmol, 0.01 eq) RuCl_2_[(*S*)-dm-segphos] [(*S*)-daipen] were added. The mixture was saturated with hydrogen for 15 min before the flask was sealed with a rubber septum and equipped with a hydrogen balloon. The mixture is stirred for 72 h before the reaction is stopped by degassing with Ar and removing volatiles under reduced pressure. The crude product is purified by recrystallization from DCM to yield 9.28 g (95%) as a white solid. ^1^H-NMR (500 MHz, CDCl_3_): δ 7.14 (t, J = 7.8 Hz, 1H), 6.86 (t, J = 2.0 Hz, 1H), 6.79 (d, J = 7.7 Hz, 1H), 6.76 – 6.70 (m, 2H), 6.69 – 6.61 (m, 2H), 4.58 (dd, J = 7.6, 5.4 Hz, 1H), 3.80 (s, 3H), 3.78 (s, 3H), 2.71 – 2.47 (m, 2H), 2.13 – 1.85 (m, 2H). ^13^C-NMR (126 MHz, CDCl_3_): δ 156.3, 148.9, 147.2, 146.1, 134.4, 129.8, 120.4, 118.2, 114.9, 112.9, 112.1, 111.5, 74.0, 56.0, 55.9, 40.3, 31.6. LC-MS: t_R_ = 9.87 min (99%), m/z: calculated = 271.13 [M+H-H_2_O]^+^, found = 271.26 [M+H-H_2_O]^+^. Chiral HPLC (30% B): t_R_ = 8.78 min (> 99%)

**(R)-3-(3,4-Dimethoxyphenyl)-1-(3-(2-morpholinoethoxy)phenyl)propan-1-ol (10):** 3.33 g (11.5 mmol, 1 eq) **9**, 2.15 g (11.5 mmol, 1 eq) 2-Chloroethylmorpholine hydrochloride, and 6.38 g (66.1 mmol, 4 eq) potassium carbonate are suspended in 50 mL MeCN. The suspension is refluxed for 24 h before the reaction is stopped by filtering off the solids and removing volatiles under reduced pressure. The crude product is purified by manual silica column (EA/TEA/MeOH 100:3:1) to yield 3.60 g (78%) as a yellow oil. ^1^H-NMR (500 MHz, CDCl_3_): δ 7.28 – 7.21 (m, 1H), 6.92 (dt, J = 6.6, 1.4 Hz, 2H), 6.83 – 6.79 (m, 1H), 6.79 – 6.77 (m, 1H), 6.75 – 6.69 (m, 2H), 4.65 (dd, J = 7.9, 5.2 Hz, 1H), 4.15 – 4.06 (m, 2H), 3.85 (s, 3H), 3.84 (s, 3H), 3.74 – 3.68 (m, 4H), 2.78 (td, J = 5.7, 1.2 Hz, 2H), 2.75 – 2.56 (m, 2H), 2.58 – 2.53 (m, 4H), 2.14 – 1.93 (m, 2H). ^13^C-NMR (126 MHz, CDCl_3_): δ 159.1, 149.0, 147.3, 146.6, 134.5, 129.6, 120.3, 118.6, 113.7, 112.3, 111.9, 111.4, 73.8, 67.0, 65.9, 57.8, 56.1, 56.0, 54.2, 40.7, 31.8. UHPLC-MS: t_R_ = 1.358 min (98%), m/z: calculated = 402.23 [M+H]^+^, found = 402.2 [M+H]^+^.

**Perfluorophenyl 2-(3,4,5-trimethoxyphenyl)acetate:** 50 g (221 mmol, 1.0 eq) 3,4,5,-Trimethoxyphenylacetic acid, 40.7 g (221 mmol, 1.0 eq) pentafluorophenol, and 4.96 g (44.2 mmol, 0.2 eq) DMAP are dissolved in 500 mL DCM and cooled to 0 °C, 46.6 g (243 mmol, 1.1 eq) EDC・HCl are added and the mixture is stirred overnight at room temperature. After quenching the reaction by adding brine, the mixture is extracted three times using DCM, the combined organic phases are washed twice with 1 M HCl, dried over MgSO_4_, and volatiles are removed under reduced pressure. The crude product is recrystallized from MeOH to yield 64.2 g (74%) as a white solid. ^1^H-NMR (500 MHz, CDCl_3_): δ 6.57 (s, 2H), 3.90 (s, 2H), 3.88 (s, 6H), 3.85 (s, 3H). ^13^C-NMR (126 MHz, CDCl_3_): δ 153.2, 136.4, 127.9, 106.4, 60.8, 56.1. LC-MS: tR = 13.43 min (99%), m/z: calculated = 393.08 [M+H]^+^, found = 393.07 [M+H]^+^.

**(S)-4-Phenyl-3-(2-(3,4,5-trimethoxyphenyl)acetyl)oxazolidin-2-one (2):** 28.19 g (173 mmol, 1.1 eq) (S)-4-Phenyloxazolidin-2-one are dissolved in 620 mL THF and cooled to −78 °C. 68.0 mL (173 mmol, 1.1 eq) *n*-butyl lithium are slowly added and the mixture is stirred for 15 min. 61.6 g (157 mmol, 1.0 eq of **Perfluorophenyl 2-(3,4,5-trimethoxyphenyl)acetate** are added and the mixture is stirred for 2 h at −78 °C before the reaction is quenched by addition of isopropyl alcohol (IPA). The mixture was extracted with EA, dried over MgSO_4_, and volatiles are removed under reduced pressure. The crude product is purified by flash column chromatography (Cy/EA gradient) to yield 40.0 g (76%) as a white solid. ^1^H-NMR (500 MHz, CDCl_3_): δ 7.37 – 7.28 (m, 3H), 7.23 – 7.15 (m, 2H), 6.45 (s, 2H), 5.43 (dd, J = 8.8, 4.0 Hz, 1H), 4.69 (t, J = 8.9 Hz, 1H), 4.30 – 4.23 (m, 2H), 4.16 (d, J = 14.8 Hz, 1H), 3.82 (s, 3H), 3.77 (s, 6H). ^13^C-NMR (126 MHz, CDCl_3_): δ 170.6, 153.7, 153.3, 138.8, 137.3, 134.1, 129.2, 128.9, 126.1, 106.8, 70.0, 61.0, 57.9, 56.2, 41.8. UHPLC-MS: t_R_ = 1.98 min (96%), m/z: calculated = 372.15 [M+H]^+^, found = 372.2 [M+H]^+^.

**(4S)-3-(2-(cyclohex-2-en-1-yl)-2-(3,4,5-trimethoxyphenyl)acetyl)-4-phenyloxazolidin-2-one:** 40.0 g (108 mmol, 1.0 eq) **2** are dissolved in 240 mL THF and cooled to 78 °C. 118 mL (118 mmol, 1.1 eq) of 1 M LiHMDS are added and the mixture is stirred for 2 h at −78 °C. 18.9 mL (162 mmol, 1.5 eq) 3-bromocyclohexene are added and the mixture is stirred for another 2 h before the reaction is quenched by addition of NH_4_Cl solution. The mixture was extracted with EA, dried over MgSO_4_, and volatiles are removed under reduced pressure. The crude product is purified by flash column chromatography (Cy/EA gradient) to yield 35.9 g (74%) as a white solid as a mixture of diastereomers. ^1^H-NMR (500 MHz, CDCl_3_): δ 7.43 – 7.37 (m, 2H), 7.36 – 7.30 (m, 3H), 6.67 (d, J = 8.7 Hz, 2H), 5.61 (dqd, J = 9.7, 4.6, 1.8 Hz, 1H), 5.36 (ddd, J = 9.2, 5.6, 3.7 Hz, 1H), 5.04 (dq, J = 10.2, 2.3 Hz, 1H), 4.83 (d, J = 11.3 Hz, 1H), 4.57 (td, J = 8.9, 3.2 Hz, 1H), 4.20 (ddd, J = 8.9, 3.8, 2.1 Hz, 1H), 3.83 (d, J = 2.8 Hz, 6H), 3.81 (d, J = 0.7 Hz, 3H), 2.80 (ddp, J = 16.1, 7.7, 2.6 Hz, 1H), 1.93 (ddq, J = 12.4, 5.9, 3.3 Hz, 2H), 1.70 – 1.58 (m, 1H), 1.49 – 1.28 (m, 2H), 1.11 (tdd, J = 12.7, 6.2, 2.6 Hz, 1H). ^13^C-NMR (126 MHz, CDCl_3_): δ 173.5, 153.5, 153.4, 153.2, 139.4, 139.4, 137.4, 132.7, 132.6, 129.4, 129.2, 129.1, 128.9, 128.8, 128.5, 128.0, 126.1, 126.0, 106.4, 106.2, 69.5, 69.5, 60.8, 58.2, 58.2, 56.2, 56.2, 53.6, 53.4, 39.7, 39.6, 27.6, 26.1, 25.3, 25.2, 21.1, 20.4. UHPLC-MS: t_R_ = 1.150 min (99%), m/z: calculated = 452.21 [M+H]^+^, found = 452.2 [M+H]^+^.

**(S)-3-((R)-2-cyclohexyl-2-(3,4,5-trimethoxyphenyl)acetyl)-4-phenyloxazolidin-2-one (4a) and (S)-3-((S)-2-Cyclohexyl-2-(3,4,5-trimethoxyphenyl)acetyl)-4-phenyloxazolidin-2-one (4b):** 23.2 g (51.4 mmol, 1.0 eq) **(4S)-3-(2-(cyclohex-2-en-1-yl)-2-(3,4,5-trimethoxyphen-yl)acetyl)-4-phenyloxazolidin-2-one** are dissolved in 220 mL MeOH and the mixture is degassed with Ar for 15 min before 2.73 g (2.57 mmol, 0.05 eq) Pd/C are added. The mixture is then saturated with hydrogen gas for 15 min before the flask is sealed with a rubber septum and equipped with a hydrogen balloon. The reaction is stirred overnight at room temperature and then stopped by degassing the solution for 15 min with Ar before filtering over silica. Volatiles are removed under reduced pressure. The crude product is purified by flash column chromatography (Cy/EA gradient) to yield **11a** 16.38 g (70%) as a white solid and **11b** 4.38 g (19%) as a white solid.

**4a:** ^1^H-NMR (500 MHz, CDCl_3_): δ 7.22 – 7.12 (m, 3H), 6.85 – 6.79 (m, 2H), 6.27 (s, 2H), 5.44 (dd, J = 9.0, 5.0 Hz, 1H), 4.72 (d, J = 10.7 Hz, 1H), 4.68 – 4.58 (m, 1H), 3.83 (s, 3H), 3.63 (s, 6H), 2.05 – 1.92 (m, 1H), 1.85 (dt, J = 12.4, 3.1 Hz, 1H), 1.70 (dt, J = 14.7, 3.9 Hz, 1H), 1.65 – 1.57 (m, 3H), 1.35 – 1.20 (m, 2H), 1.20 – 1.07 (m, 2H), 1.02 (tdd, J = 12.4, 10.8, 3.6 Hz, 1H), 0.82 (qd, J = 11.8, 3.0 Hz, 1H). ^13^C-NMR (126 MHz, CDCl3): δ 172.7, 153.3, 153.0, 138.4, 137.1, 132.1, 128.8, 128.5, 125.9, 106.0, 69.5, 60.9, 57.9, 56.0, 55.9, 39.8, 32.4, 30.3, 26.5, 26.1, 26.1. UHPLC-MS: t_R_ = 1.186 min (99%), m/z: calculated = 454.23 [M+H]^+^, found = 454.2 [M+H]^+^.

**4b:** ^1^H-NMR (500 MHz, CDCl_3_): δ 7.46 – 7.26 (m, 5H), 5.36 (dd, J = 8.8, 3.6 Hz, 1H), 4.79 (d, J = 10.8 Hz, 1H), 4.58 (t, J = 8.8 Hz, 1H), 4.21 (dd, J = 8.9, 3.6 Hz, 1H), 3.84 (s, 6H), 3.82 (s, 3H), 2.08 – 1.89 (m, 1H), 1.58 (d, J = 16.1 Hz, 4H), 1.36 – 1.20 (m, 3H), 1.09 (d, J = 8.3 Hz, 2H), 1.01 – 0.62 (m, 1H). ^13^C-NMR (126 MHz, CDCl_3_): δ 174.0, 153.7, 153.1, 139.5, 137.2, 133.2, 129.3, 128.9, 126.0, 106.2, 69.5, 61.0, 58.3, 56.2, 54.7, 42.2, 31.5, 30.3, 26.4, 26.0, 25.9. UHPLC-MS: t_R_ = 1.238 min (99%), m/z: calculated = 454.23 [M+H]^+^, found = 454.2 [M+H]^+^.

**(S)-2-Cyclohexyl-2-(3,4,5-trimethoxyphenyl)acetic acid:** 16.38 g (36.1 mmol, 1.0 eq) **4b** are dissolved in 160 mLTHF and added to a mixture of 2.59 g (108 mmol, 3.0 eq) LiOH and 18.4 mL (181 mmol, 5.0 eq) H_2_O_2_ dissolved in 80 mL water. The mixture is stirred overnight at room temperature before the reaction is quenched by addition of 1 M HCl. The mixture is extracted with ether, dried over MgSO_4_, and the solvent is removed under reduced pressure. The crude product is purified by flash column chromatography (Cy/EA+1% of formic acid gradient) to yield 9.7 g (87%) as a pale yellow solid. ^1^H-NMR (500 MHz, CDCl_3_): δ 6.54 (s, 2H), 3.84 (s, 6H), 3.82 (s, 3H), 3.12 (d, J = 10.7 Hz, 1H), 1.95 (tt, J = 11.0, 3.3 Hz, 1H), 1.92 – 1.86 (m, 1H), 1.79 – 1.71 (m, 1H), 1.64 (dq, J = 8.0, 3.9 Hz, 2H), 1.43 – 1.34 (m, 1H), 1.34 – 1.24 (m, 1H), 1.22 – 1.13 (m, 2H), 1.07 (tdd, J = 12.6, 10.8, 3.5 Hz, 1H), 0.81 – 0.69 (m, 1H). ^13^C-NMR (126 MHz, CDCl_3_): δ 180.0, 153.3, 137.5, 133.0, 105.8, 60.9, 59.1, 56.3, 41.0, 32.0, 30.4, 26.4, 26.0. UHPLC-MS: t_R_ = 1.863 min (99%), m/z: calculated = 309.2 [M+H]^+^, found = 309.2 [M+H]^+^.

**(R)-2-Cyclohexyl-2-(3,4,5-trimethoxyphenyl)acetic acid (5):** 4.34 g (9.57 mmol, 1.0 eq) **4a** are dissolved in 80 mL THF. The mixture is cooled to 0 °C and 0.69 g (28.7 mmol, 3.0 eq) LiOH and 4.89 mL (47.9 mmol, 5.0 eq) H_2_O_2_ dissolved in 50 mL are added. The mixture is stirred overnight at room temperature before the reaction is quenched by addition of diluted HCl. The mixture is extracted with EA, dried with MgSO_4_, and volatiles are removed under reduced pressure. The crude product is purified by flash column chromatography (DCM/MeOH + 1% FA) to yield 2.03 g (69%) as a white solid. ^1^H-NMR (500 MHz, CDCl_3_): δ 6.54 (s, 2H), 3.84 (s, 6H), 3.82 (s, 3H), 3.12 (d, J = 10.7 Hz, 1H), 2.02 – 1.85 (m, 2H), 1.79 – 1.71 (m, 1H), 1.68 – 1.58 (m, 2H), 1.41 – 1.23 (m, 2H), 1.22 – 1.01 (m, 3H), 0.81 – 0.69 (m, 1H). ^13^C-NMR (126 MHz, CDCl_3_): δ 179.9, 153.3, 137.5, 133.0, 129.3, 126.1, 105.8, 61.0, 59.1, 56.3, 41.0, 32.1, 30.4, 26.4, 26.1. UHPLC-MS: t_R_ = 0.492 min (97%), m/z: calculated = 309.18 [M+H]+, found = 309.2 [M+H]^+^.

**(S)-1-Tert-butyl 2-((R)-3-(3,4-dimethoxyphenyl)-1-(3-(2-morpholinoethoxy)phenyl)pro-pyl) piperidine-1,2-dicarboxylate:** 31.3 g (77.8 mmol, 1.0 eq) **10**, 18.74 g (81.7 mmol, 1.05 eq) (*S*)-N-Boc-pipecolate, and 1.90 g (15.6 mmol, 0.2 eq) DMAP are dissolved in 350 mL DCM. The mixture is cooled to 0 °C and 15.7 g (81.7 mmol, 1.05 eq) EDC・HCl are added. The mixture is stirred overnight at room temperature before the reaction is quenched by addition of brine. The mixture is extracted with DCM, dried with MgSO_4_, and volatiles are removed under reduced pressure. The crude product is purified by flash column chromatography (Cy/EA + 3% TEA) to yield 42.2 g (97%) as a pale yellow oil. ^1^H-NMR (500 MHz, CDCl_3_): δ 7.24 (t, J = 8.2 Hz, 2H), 6.91 (d, J = 7.8 Hz, 2H), 6.87 (s, 2H), 6.82 (d, J = 8.2 Hz, 1H), 6.77 (d, J = 8.1 Hz, 1H), 6.67 (q, J = 6.6 Hz, 3H), 5.76 (s, 1H), 5.04 – 4.89 (m, 1H), 4.75 (d, J = 6.0 Hz, 1H), 4.10 (p, J = 6.3 Hz, 5H), 4.06 – 3.90 (m, 1H), 3.84 (d, J = 2.5 Hz, 9H), 3.80 – 3.68 (m, 7H), 2.79 (t, J = 5.7 Hz, 3H), 2.67 – 2.42 (m, 4H), 2.25 (dd, J = 26.2, 13.2 Hz, 4H), 2.04 (d, J = 8.3 Hz, 4H), 1.77 – 1.55 (m, 5H), 1.47 (s, 6H), 1.36 (d, J = 7.2 Hz, 7H), 1.22 – 1.02 (m, 1H). ^13^C-NMR (126 MHz, CDCl_3_): δ 171.5, 171.2, 158.9, 155.5, 149.0, 147.5, 141.9, 133.8, 133.6, 129.7, 120.3, 119.2, 114.2, 114.0, 113.1, 111.8, 111.4, 80.0, 76.2, 67.0, 65.9, 60.5, 57.8, 56.0, 55.9, 55.9, 55.1, 54.2, 54.0, 49.2, 42.3, 41.2, 38.4, 38.2, 34.1, 31.5, 31.2, 28.5, 28.4, 26.9, 25.7, 25.1, 25.0, 24.7, 21.1, 21.0, 20.8, 14.3. UHPLC-MS: t_R_ = 2.072 min (99%), m/z: calculated = 613.35 [M+H]^+^, found = 613.4 [M+H]^+^.

**(S)-(R)-3-(3,4-Dimethoxyphenyl)-1-(3-(2-morpholinoethoxy)phenyl)propyl**

**piperidine-2-carboxylate:** 42.2 g (68.9 mmol, 1.0 eq) **(S)-1-Tert-butyl 2-((R)-3-(3,4-dimethoxyphenyl)-1-(3-(2-morpholinoethoxy)phenyl)pro-pyl) piperidine-1,2-dicarboxylate** are dissolved in 40 mL of DCM and 20 mL TFA. The mixture is stirred for 2 h at room temperature before the reaction is quenched by addition of NaHCO_3_. The mixture is extracted with DCM, dried with MgSO_4_, and volatiles are removed under reduced pressure. The crude product (35.3 g quant.) is used without further purification. ^1^H-NMR (500 MHz, CDCl_3_): δ 7.22 – 7.15 (m, 1H), 6.86 (d, J = 7.6 Hz, 1H), 6.82 (d, J = 2.5 Hz, 1H), 6.77 (dd, J = 8.2, 2.5 Hz, 1H), 6.73 (d, J = 8.1 Hz, 1H), 6.65 – 6.56 (m, 2H), 5.70 (dd, J = 8.1, 5.5 Hz, 1H), 4.04 (t, J = 5.6 Hz, 2H), 3.80 (s, 3H), 3.79 (s, 3H), 3.68 (t, J = 4.5 Hz, 4H), 3.32 (dd, J = 10.1, 3.3 Hz, 1H), 3.02 (dt, J = 12.2, 3.8 Hz, 1H), 2.74 (t, J = 5.8 Hz, 2H), 2.65 – 2.54 (m, 1H), 2.54 – 2.44 (m, 6H), 2.18 (ddt, J = 14.7, 9.0, 4.2 Hz, 1H), 2.05 – 1.94 (m, 2H), 1.75 (dt, J = 8.5, 4.8 Hz, 1H), 1.59 – 1.48 (m, 2H), 1.47 – 1.36 (m, 2H), 1.16 – 0.96 (m, 1H). ^13^C-NMR (126 MHz, CDCl_3_): δ 172.8, 158.8, 148.8, 147.3, 141.8, 133.6, 129.5, 120.1, 120.1, 119.0, 119.0, 113.8, 113.0, 111.7, 111.7, 111.3, 75.5, 67.0, 66.9, 65.7, 58.7, 57.6, 55.9, 55.8, 55.8, 54.1, 52.9, 45.9, 45.6, 38.0, 33.9, 31.3, 31.3, 29.3, 25.8, 25.7, 25.0, 24.1, 9.3, 8.1. UHPLC-MS: t_R_ = 1.302 min (98%), m/z: calculated = 513.30 [M+H]^+^, found = 513.2 [M+H]^+^.

**(S)-(R)-3-(3,4-Dimethoxyphenyl)-1-(3-(2-morpholinoethoxy)phenyl)propyl**

**1-((S)-2-cyclohexyl-2-(3,4,5-trimethoxyphenyl)acetyl)piperidine-2-carboxylate (SAFit2):** 13.4 g (43.3 mmol, 1.0 eq) **(S)-2-Cyclohexyl-2-(3,4,5-trimethoxyphenyl)acetic acid**, 18.1 g (47.6 mmol, 1.1 eq) HATU, and 30.2 mL (173 mmol, 4.0 eq) DiPEA are dissolved in 20 mL of DMF. The mixture is stirred for 30 min at room temperature before 33.3 g (64.9 mmol, 1.5 eq) **(S)-(R)-3-(3,4-Dimethoxyphenyl)-1-(3-(2-morpholinoethoxy)phenyl)propyl piperidine-2-carboxylate** dissolved in 250 mL DCM are added. The mixture is stirred overnight at room temperature before the reaction is quenched by addition of brine. The mixture is extracted with DCM, dried with MgSO_4_, and volatiles are removed under reduced pressure. The crude product is purified by preparative HPLC and flash column chromatography (Cy/EA+3% TEA) to yield 19.5 g (56%) as a white solid. ^1^H-NMR (700 MHz, CDCl_3_): δ 7.28 (t, J = 7.9 Hz, 0H), 7.09 (t, J = 7.9 Hz, 1H), 6.95 (d, J = 7.6 Hz, 0H), 6.90 (t, J = 2.1 Hz, 0H), 6.88 – 6.84 (m, 0H), 6.78 (d, J = 8.2 Hz, 0H), 6.76 – 6.73 (m, 2H), 6.69 (t, J = 2.0 Hz, 1H), 6.68 (dd, J = 8.2, 2.0 Hz, 0H), 6.65 (d, J = 2.0 Hz, 0H), 6.63 – 6.60 (m, 2H), 6.47 (s, 2H), 6.41 (s, 1H), 6.39 (d, J = 7.6 Hz, 1H), 5.79 (t, J = 7.0 Hz, 0H), 5.55 (dd, J = 8.2, 5.5 Hz, 1H), 5.46 (d, J = 5.6 Hz, 1H), 4.71 (d, J = 5.8 Hz, 0H), 4.55 (d, J = 13.6 Hz, 0H), 4.14 – 4.08 (m, 1H), 4.06 (t, J = 5.7 Hz, 2H), 3.93 (d, J = 13.6 Hz, 1H), 3.85 (s, 1H), 3.84 (s, 1H), 3.84 (s, 3H), 3.83 (s, 5H), 3.82 (s, 1H), 3.76 (s, 3H), 3.71 (t, J = 4.7 Hz, 6H), 3.70 (s, 5H), 3.36 (d, J = 9.8 Hz, 1H), 2.96 (d, J = 9.7 Hz, 0H), 2.81 – 2.78 (m, 1H), 2.77 (t, J = 5.7 Hz, 2H), 2.65 – 2.52 (m, 6H), 2.46 (ddd, J = 14.4, 9.4, 5.3 Hz, 1H), 2.37 (ddd, J = 13.9, 9.1, 7.0 Hz, 1H), 2.28 (d, J = 13.7 Hz, 1H), 2.13 – 2.04 (m, 2H), 2.03 (s, 0H), 1.95 (dtd, J = 14.0, 8.7, 5.3 Hz, 1H), 1.87 (d, J = 11.9 Hz, 1H), 1.85 – 1.75 (m, 1H), 1.72 – 1.64 (m, 2H), 1.64 – 1.61 (m, 2H), 1.61 – 1.52 (m, 2H), 1.45 – 1.37 (m, 1H), 1.36 – 1.27 (m, 2H), 1.27 – 1.21 (m, 1H), 1.19 – 1.07 (m, 3H), 0.99 (t, J = 12.6 Hz, 0H), 0.89 (qd, J = 12.5, 3.5 Hz, 1H), 0.78 – 0.71 (m, 1H), 0.64 – 0.50 (m, 1H). ^13^C-NMR (176 MHz, CDCl_3_): δ 172.4, 172.3, 171.2, 170.7, 170.6, 159.1, 158.7, 153.4, 153.1, 149.1, 148.9, 147.6, 147.4, 141.9, 141.6, 137.1, 136.9, 134.4, 133.7, 133.6, 133.4, 129.9, 129.6, 120.3, 120.2, 119.2, 118.5, 114.2, 113.8, 113.4, 113.2, 111.9, 111.8, 111.5, 111.4, 105.9, 105.4, 76.9, 75.7, 67.0, 67.0, 65.9, 65.8, 61.0, 60.9, 60.5, 57.8, 56.4, 56.1, 56.0, 56.0, 56.0, 56.0, 55.9, 55.9, 55.8, 55.1, 54.2, 52.1, 43.7, 41.5, 41.2, 39.5, 38.1, 37.9, 33.1, 32.9, 31.6, 31.1, 30.8, 30.6, 26.9, 26.7, 26.5, 26.3, 26.3, 26.2, 25.6, 24.4, 21.1, 21.1, 20.8, 14.3. UHPLC-MS: t_R_ = 1.614 min (> 99%), m/z: calculated = 803.45 [M+H]^+^, found = 803.4 [M+H]^+^.

**(R)-1-tert-Butyl 2-((R)-3-(3,4-dimethoxyphenyl)-1-(3-(2-morpholinoethoxy)phenyl)prop-yl) piperidine-1,2-dicarboxylate:** 4.00 g (10.0 mmol, 1.0 eq) **10**, 2.40 g (10.5 mmol, 1.05 eq) *R*-N-Boc-pipecolate, and 0.24 g (2.0 mmol, 0.2 eq) DMAP are dissolved in 50 mL DCM. The mixture is cooled to 0 °C and 2.01 g (10.5 mmol, 1.05 eq) DCC are added. The mixture is stirred overnight at room temperature before the reaction is quenched by addition of brine. The mixture is extracted with DCM, dried with MgSO_4_, and volatiles are removed under reduced pressure. The crude product is purified by manual column chromatography (EA/TEA/MeOH 100:3:1) to yield 4.78 g (78%) as a white solid. ^1^H-NMR (500 MHz, CDCl_3_): δ 7.23 (td, J = 7.9, 5.2 Hz, 1H), 6.90 (q, J = 6.9 Hz, 1H), 6.85 (d, J = 1.8 Hz, 1H), 6.84 – 6.79 (m, 2H), 6.77 (d, J = 8.1 Hz, 1H), 6.68 (dd, J = 8.0, 2.0 Hz, 1H), 6.66 (d, J = 2.0 Hz, 1H), 5.74 (dt, J = 32.3, 6.9 Hz, 1H), 4.98 – 4.88 (m, 1H), 4.77 (s, 0H), 4.09 (t, J = 5.8 Hz, 2H), 3.88 – 3.82 (m, 6H), 3.75 – 3.69 (m, 4H), 2.93 (dt, J = 34.6, 13.1 Hz, 1H), 2.78 (t, J = 5.7 Hz, 2H), 2.66 – 2.47 (m, 6H), 2.22 (qd, J = 15.3, 10.0 Hz, 3H), 2.04 (ddd, J = 16.2, 12.9, 6.4 Hz, 1H), 1.72 – 1.52 (m, 3H), 1.43 (d, J = 32.0 Hz, 11H), 1.22 – 1.02 (m, 1H). ^13^C-NMR (126 MHz, CDCl_3_): δ 171.5, 171.3, 159.0, 156.1, 155.4, 149.0, 147.5, 142.3, 141.9, 133.8, 133.7, 129.6, 120.3, 118.8, 114.1, 113.9, 113.2, 112.6, 111.9, 111.5, 80.1, 80.0, 76.2, 76.1, 67.0, 65.9, 57.8, 56.0, 56.0, 55.0, 54.2, 53.9, 42.3, 41.2, 38.5, 38.4, 31.6, 31.4, 28.5, 26.9, 26.8, 24.9, 24.7, 20.8, 20.7. UHPLC-MS: t_R_ = 1.786 min (97%), m/z: calculated = 613.35 [M+H]^+^, found = 613.2 [M+H]^+^.

**(R)-(R)-3-(3,4-Dimethoxyphenyl)-1-(3-(2-morpholinoethoxy)phenyl)propyl-**

**piperidine-2-carboxylate (11):** 4.78 g (7.8 mmol, 1.0 eq) **(R)-1-tert-Butyl 2-((R)-3-(3,4-dimethoxyphenyl)-1-(3-(2-morpholinoethoxy)phenyl)prop-yl) piperidine-1,2-dicarboxylate** is dissolved in 24 mL DCM and 12 mL TFA. The mixture is stirred for 1.5 h at room temperature before the mixture was concentrated under reduced pressure and the reaction was neutralized with sodium carbonate. The mixture is extracted with DCM, dried with MgSO_4_, and volatiles are removed under reduced pressure. The crude product (4.0 g quant.) is used without further purification. ^1^H-NMR (500 MHz, CDCl_3_): δ 7.22 (td, J = 7.9, 1.4 Hz, 1H), 6.89 (dt, J = 7.7, 1.3 Hz, 1H), 6.85 (dd, J = 2.6, 1.6 Hz, 1H), 6.80 (ddd, J = 8.2, 2.6, 1.0 Hz, 1H), 6.76 (dd, J = 7.9, 1.4 Hz, 1H), 6.69 – 6.62 (m, 2H), 5.74 (dd, J = 7.9, 5.8 Hz, 1H), 4.08 (td, J = 5.8, 2.3 Hz, 2H), 3.84 (d, J = 1.3 Hz, 3H), 3.83 (d, J = 1.3 Hz, 3H), 3.74 – 3.69 (m, 4H), 3.42 – 3.32 (m, 1H), 3.06 (dt, J = 12.2, 3.7 Hz, 1H), 2.77 (td, J = 5.8, 1.4 Hz, 2H), 2.69 – 2.60 (m, 1H), 2.59 – 2.47 (m, 6H), 2.28 – 2.17 (m, 1H), 2.10 – 1.99 (m, 2H), 1.98 – 1.91 (m, 1H), 1.74 (dq, J = 7.7, 3.4 Hz, 1H), 1.60 – 1.53 (m, 1H), 1.51 – 1.33 (m, 3H). ^13^C-NMR (126 MHz, CDCl_3_): δ 172.8, 158.9, 148.9, 147.4, 142.0, 133.7, 129.6, 120.2, 119.0, 113.9, 113.2, 113.1, 111.8, 111.4, 75.7, 67.0, 65.9, 58.7, 57.7, 56.0, 55.9, 54.2, 45.8, 37.9, 31.4, 29.3, 26.0, 24.1. UHPLC-MS: t_R_ = 1.256 min (97%), m/z: calculated = 513.30 [M+H]^+^, found = 513.2 [M+H]^+^.

**(R)-(R)-3-(3,4-Dimethoxyphenyl)-1-(3-(2-morpholinoethoxy)phenyl)propyl**

**1-((R)-2-cyclohexyl-2-(3,4,5-trimethoxyphenyl)acetyl)piperidine-2-carboxylate (ddSAFit, 12):** 2.03 g (6.6 mmol, 1.0 eq) **4a**, and 2.63 g (6.9 mmol, 1.05 eq) HATU, and 4.6 mL (26.3 mmol, 4.0 eq) DiPEA are dissolved in 10 mL of DMF. The mixture is stirred for 30 min at room temperature before 4.0 g (7.8 mmol, 1.2 eq) **11** dissolved in 50 mL DCM are added. The mixture is stirred overnight at room temperature before the reaction is quenched by addition of brine. The mixture is extracted with EA, dried with MgSO_4_, and volatiles are removed under reduced pressure. The crude product is purified by preparative HPLC and flash column chromatography (Cy/EA+3% TEA) to yield 4.12 g (78%) as a white solid. ^1^H-NMR (700 MHz, CDCl_3_): δ 7.29 – 7.25 (m, 0H), 7.18 (t, J = 8.0 Hz, 1H), 6.94 (d, J = 7.6 Hz, 0H), 6.93 – 6.83 (m, 1H), 6.80 – 6.73 (m, 3H), 6.71 – 6.64 (m, 1H), 6.62 – 6.56 (m, 2H), 6.49 (s, 1H), 6.41 (s, 1H), 5.78 (dd, J = 7.9, 6.2 Hz, 0H), 5.55 (dd, J = 8.4, 5.2 Hz, 1H), 5.43 – 5.40 (m, 1H), 4.73 (d, J = 5.6 Hz, 0H), 4.66 (d, J = 13.6 Hz, 0H), 4.12 – 4.02 (m, 2H), 3.96 (d, J = 13.8 Hz, 1H), 3.87 – 3.80 (m, 9H), 3.77 (s, 6H), 3.74 – 3.69 (m, 4H), 3.38 (d, J = 9.8 Hz, 1H), 3.10 (d, J = 9.6 Hz, 0H), 2.97 (td, J = 13.4, 2.9 Hz, 1H), 2.78 (dt, J = 17.0, 5.7 Hz, 2H), 2.65 – 2.51 (m, 5H), 2.47 (ddd, J = 14.3, 9.0, 5.5 Hz, 1H), 2.36 (dt, J = 14.6, 7.8 Hz, 1H), 2.26 (dd, J = 12.0, 4.1 Hz, 1H), 2.15 – 2.02 (m, 2H), 1.98 – 1.85 (m, 3H), 1.74 – 1.50 (m, 5H), 1.43 (q, J = 12.9 Hz, 1H), 1.37 – 1.08 (m, 4H), 1.06 – 0.97 (m, 0H), 0.92 (qd, J = 12.4, 3.5 Hz, 1H), 0.82 – 0.55 (m, 1H). ^13^C-NMR (176 MHz, CDCl_3_): δ 172.8, 171.9, 170.6, 170.5, 159.0, 153.4, 153.1, 149.1, 149.0, 147.6, 147.4, 142.3, 141.3, 137.1, 136.9, 134.3, 133.6, 133.6, 133.4, 129.8, 129.7, 120.3, 120.3, 120.2, 119.3, 118.5, 114.1, 113.8, 113.6, 112.7, 111.9, 111.8, 111.5, 111.4, 105.9, 105.4, 77.0, 75.7, 67.0, 67.0, 66.0, 65.9, 61.0, 60.8, 57.8, 57.8, 56.3, 56.2, 56.1, 56.1, 55.9, 55.8, 55.1, 54.2, 54.2, 52.3, 43.7, 41.6, 41.2, 39.7, 38.2, 37.9, 33.2, 32.9, 31.6, 31.3, 30.8, 30.8, 26.9, 26.8, 26.7, 26.7, 26.4, 26.3, 26.3, 26.3, 25.6, 24.6, 21.0, 20.8. UHPLC-MS: t_R_ = 1.881 min (> 99%), m/z: calculated = 803.45 [M+H]^+^, found = 803.4 [M+H]^+^. HRMS: m/z: calculated =803.4477, found = 803.4461 [M+H]^+^ (1.99 ppm error)
